# An S-Locus Independent Pollen Factor Confers Self-Compatibility in ‘Katy’ Apricot

**DOI:** 10.1371/journal.pone.0053947

**Published:** 2013-01-14

**Authors:** Elena Zuriaga, Juan V. Muñoz-Sanz, Laura Molina, Ana D. Gisbert, María L. Badenes, Carlos Romero

**Affiliations:** Fruit Tree Breeding Department, Instituto Valenciano de Investigaciones Agrarias, Moncada, Valencia, Spain; Washington State University, United States of America

## Abstract

Loss of pollen-*S* function in *Prunus* self-compatible cultivars has been mostly associated with deletions or insertions in the *S*-haplotype-specific F-box (*SFB*) genes. However, self-compatible pollen-part mutants defective for non-*S*-locus factors have also been found, for instance, in the apricot (*Prunus armeniaca*) cv. ‘Canino’. In the present study, we report the genetic and molecular analysis of another self-compatible apricot cv. termed ‘Katy’. *S*-genotype of ‘Katy’ was determined as *S*_1_ *S*_2_ and *S-RNase* PCR-typing of selfing and outcrossing populations from ‘Katy’ showed that pollen gametes bearing either the *S*_1_- or the *S*_2_-haplotype were able to overcome self-incompatibility (SI) barriers. Sequence analyses showed no SNP or indel affecting the *SFB*_1_ and *SFB*_2_ alleles from ‘Katy’ and, moreover, no evidence of pollen-*S* duplication was found. As a whole, the obtained results are compatible with the hypothesis that the loss-of-function of a *S*-locus unlinked factor gametophytically expressed in pollen (*M’*-locus) leads to SI breakdown in ‘Katy’. A mapping strategy based on segregation distortion loci mapped the *M’*-locus within an interval of 9.4 cM at the distal end of chr.3 corresponding to ∼1.29 Mb in the peach (*Prunus persica*) genome. Interestingly, pollen-part mutations (PPMs) causing self-compatibility (SC) in the apricot cvs. ‘Canino’ and ‘Katy’ are located within an overlapping region of ∼273 Kb in chr.3. No evidence is yet available to discern if they affect the same gene or not, but molecular markers seem to indicate that both cultivars are genetically unrelated suggesting that every PPM may have arisen independently. Further research will be necessary to reveal the precise nature of ‘Katy’ PPM, but fine-mapping already enables SC marker-assisted selection and paves the way for future positional cloning of the underlying gene.

## Introduction

Gametophytic self-incompatibility (GSI) is a widespread mechanism in the plant kingdom that prevents inbreeding [1]. In Solanaceae, Plantaginaceae and Rosaceae GSI is controlled by the *S*-locus that contains at least two genes coding for S-RNase and F-box proteins. S-RNases are style-specific expressed and their ribonuclease activity is essential for self-pollen rejection [2]–[4]. In turn, the *S*-locus F-box proteins (*SLF* or *SFB*) are the pollen *S*-determinants [5]–[7]. Evidence accumulated in *Petunia* and *Antirrhinum* supports a model in which SLFs are components of a SCF E3 ubiquitin ligase complex that interacts with non-self S-RNases leading to their ubiquitination and degradation by the 26S proteasome proteolytic pathway [8], [9]. Alternately, the compartmentalization model proposed by Goldraij *et al.* [10] in *Nicotiana* explains the resistance to non-self S-RNases by their sequestration in vacuolar compartments of pollen compatible tubes. A hypothetical S-RNase endosome sorting model involving both S-RNase degradation and compartmentalization has been recently proposed [11], but many pieces of the puzzle remain elusive.

Spontaneous and induced self-compatible mutants have been particularly important to support *S-RNase* and *S*-locus *F-box* genes as the *S*-determinants in *Prunus* (Rosaceae) since other functional approaches based on transgenic experiments are seriously hindered in this genus. For instance, a *Mu-*like element insertion upstream of the *S*_6m_-*RNase* in sour cherry (*Prunus cerasus*) [12] and a similar mutation in the Japanese plum (*Prunus salicina*) *S* ^e^-*RNase* [13] reduce the *S-RNase* expression level leading to a insufficient accumulation of S-RNase in the pistil which breaks the rejection mechanism. Modifications affecting the S-RNase structure and conferring self-compatibility (SC) have also been found in peach (*Prunus persica*) where the S^2m^-RNase shows a reduced stability as a consequence of the cysteine residue replacement by a tyrosine in the C5 domain [14]. Regarding the pollen-part mutations (PPM), self-compatible mutants with non-functional *SFB* genes have been identified in sweet cherry (*Prunus avium*) [15]–[17], apricot (*Prunus armeniaca*) [18], sour cherry [19], Japanese apricot (*Prunus mume*) [15] and peach [14], supporting their role as the pollen-*S* determinants in this genus. In most of these cases, the self-compatible phenotype was associated with indels in the *SFB* codifying region causing a frame-shift in translation that produces a non-functional truncated protein [20]. This seems to be a specific feature of the S-RNase based GSI system operating in *Prunu*s, since in Solanaceae the only pollen-side mutations found to cause SC are due to the *S*-heteroallelic pollen effect [21]. Therefore, *SLF* mutations were initially suggested to confer SI or lethality, but recent findings provide an alternative explanation since in the non-self recognition by multiple factors SI system, shown to operate in Solanaceae [22] and *Pyrus* (Rosaceae) [23], the loss of pollen-*S* function does not lead to SC. In contrast, all loss-of-function mutations found in *Prunus SFB* cause SC which may support differences in the self-recognition mechanism where the SFB target would be an S-RNase ‘inhibitor’ instead of the S-RNase itself [24]. Nevertheless, even considering the discrepancies, major similarities (i.e. *S*-*RNase* and *SLF/SFB* as *S*-specificity determinants) are still more striking and the model as a whole might be preserved across families [25].

As reported above, SC accessions found in Rosaceae are mostly related to mutations in pistil and pollen *S*-locus determinants [20]. However, mutations in non *S*-locus factors have also been associated with SC in sweet cherry [26], almond (*Prunus amygdalus*) [27] and diploid strawberries (*Fragaria spp*.) [28]. Genetic evidence for *S*-locus unlinked factors required for GSI, also called modifier genes, was previoulsy accumulated in Solanaceae. For instance, Ai *et al.* [29] showed that the self-compatible *Petunia hybrida* cv. ‘Strawberry Daddy’ (*S*_O_ *S*_X_) accumulates a non-functional *S*-allele (*S*_O_) and a stylar mutation in an additional factor necessary for SI. Later studies in *Nicotiana* revealed that the so called 4936 stylar factor is also required for SI [30]. Moreover, mutations in modifier loci affecting the pollen-*S* function have been suggested to explain SI breakdown in *Solanum tuberosum* [31] and *Petunia axillaris* [32]. More intriguing is the behaviour of the PPM found in *Solanum chacoense* that predicts a *S*-locus inhibitor (*Sli*) gene acting as a single dominant factor that displays sporophytic inhibition of SI [33], [34]. More recently, some stylar modifier factors have been identified and successfully cloned in *Nicotiana*, such as the small asparagine-rich protein HT-B [35], the 120 kDa glycoprotein [36] and the Kunitz-type proteinase inhibitor NaStEP [37] but their role in SI still has not been completely elucidated. Pollen modifier factors have also been identified in the Solanaceae, such as the *Petunia* pollen-expressed Skp1-like protein PhSSK1 proposed to be acting as adaptor in the SCF complex [38]. Interestingly, Matsumoto *et al.* [39] have identified a similar SFB-interacting Skp1-like protein (PavSSK1) in sweet cherry and suggest that it could also be a functional component of the SCF complex. Nevertheless, the identification of additional GSI modifier factors will be necessary to dissect completely the underlying mechanism in *Prunus*.

In apricot, the cv. ‘Canino’ (*S*_2_ *S*_C_ *Mm*) was found to contain two different mutations conferring SC, an insertion in the *SFB*_C_ gene that produces an SFB_C_ truncated protein and a mutation in a modifier gene (*m*) unlinked to the *S*-locus, both independently causing the loss of pollen-*S* function [18], [40]. In this work, we have analyzed the self-compatible apricot cv. ‘Katy’ using genetic and molecular approaches, and the compiled evidence suggest that the loss of function of an *S*-locus unlinked factor (*M*’-locus) is also involved in pollen-*S* function breakdown in this case. According to the current knowledge on GSI in *Prunus* the possible roles for the mutated modifier gene are discussed. In addition, we have paved the way for future positional cloning of the ‘Katy’ pollen-part modifier gene by fine-mapping the *M’*-locus to the distal part of apricot chr. 3. Macro- and micro-synteny of this region has been studied by comparing with the *M*-locus in ‘Canino’ and by analyzing the ORFs comprised in the peach syntenic region according to the peach genome v1.0 (International Peach Genome Initiative - IPGI; http://www.rosaceae.org/peach/genome).

## Results

### ‘Katy’ is an Apricot Self-compatible Cultivar with *S*-genotype *S*_1_ *S*_2_

**Figure 1:**
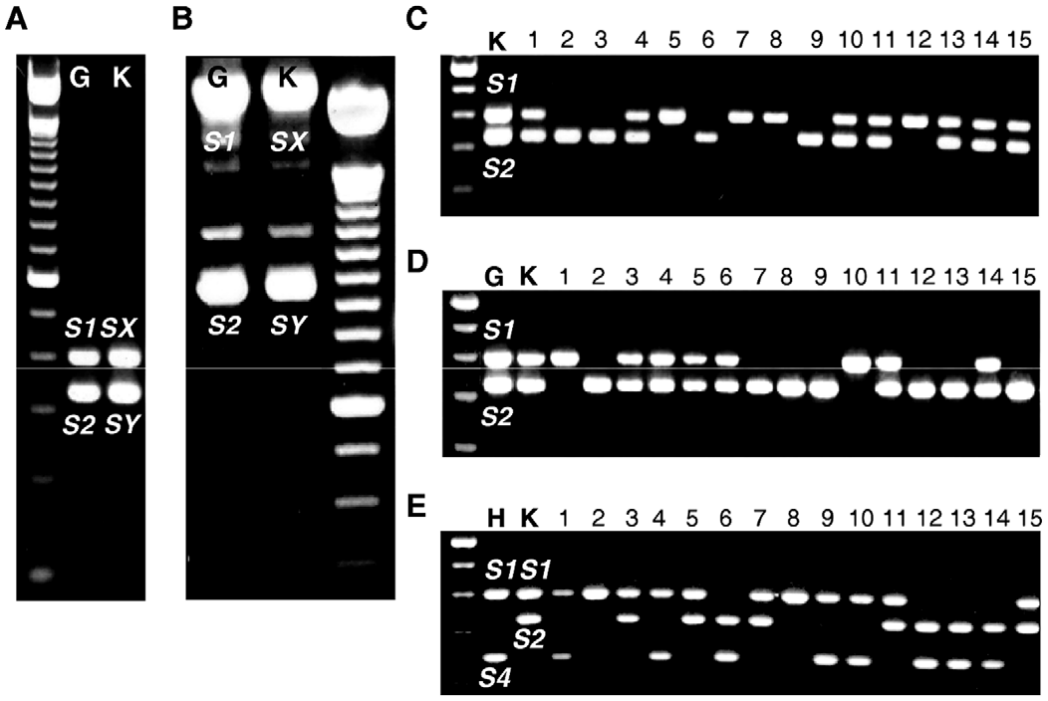
Determination of the ‘Katy’ *S*-genotype and analysis of *S*-alleles segregation in selfing and outcrossing populations derived from ‘Katy’. PCR amplification of apricot genomic DNA with consensus primers for the 1^st^ (A) and 2^nd^ (B) *S-RNase* intron. Samples in (A) and (B) are as follows: (G) Goldrich (*S*_1_ *S*_2_) and (K) Katy (*S*_x_ *S*_y_). *S*-RNase allele fragments PCR-amplified with SRc-F/SRc-R primers from the ‘K×K’ (C), ‘G×K’ (D) and ‘H×K’ (E) progenies. Samples are as follows: (K) Katy (*S*_1_ *S*_2_), (G) Goldrich (*S*_1_ *S*_2_) and (H) Harcot (*S*_1_ *S*_4_) and 15 seedlings from each cross.

‘Katy’ is an apricot variety developed by Zaigeŕs Genetics (Modesto, CA, USA) and reported as self-fruitful [41]. In this study, SC of this cultivar was confirmed by self-pollination in the field (Table 1). To determine the *S*-genotype of ‘Katy’, fragments containing the first intron of the *S-RNases* were PCR-amplified using the SRc-F/SRc-R primers (Figure 1A). These fragments were assigned to *S*_1_ and *S*_2_-alleles by comparison with known *S*-genotypes, following the nomenclature established by Burgos *et al.* [42]. This *S*-genotype was confirmed by the amplification of the second intron using the primers Pru-C2/Pru-C4R [43] since fragment sizes obtained were coincident with those expected for the *S*_1_ and *S*_2_-alleles (Figure 1B). In addition, PCR-amplified fragments spanning the first intron, were sequenced and compared with GenBank accessions, being identical to the already identified *Prunus armeniaca S*-RNases 1 and 2. The alignment of their deduced amino acid sequences (44 aa) showed the presence of the C1 and C2 *Prunus S*-RNase conserved domains along with the hypervariable region HV1 located between them [44].

**Table 1:** Segregation of the *S-RNase* alleles in progenies of self-pollinations and outcrosses performed with the self-compatible cultivar ‘Katy’.

Seed parent(*S*-genotype)^a^	Pollen parent(*S*-genotype)	Populationname	N^c^	*S*_1_ *S*_C_	*S*_2_ *S*_C_	*S*_1_ *S*_2_	*S*_2_ *S*_2_	*S*_1_ *S*_1_	*S*_1_ *S*_4_	*S*_2_ *S*_4_	Exp.Ratio^d^	?^ 2e^ *P*-value
Katy (*S*_1_ *S*_2_)	Katy (*S*_1_ *S*_2_)	‘K×K’^b^	94	–	–	45	33	16	–	–	2∶1:1	6.32 (0.04)
Katy (*S*_1_ *S*_2_)	Goldrich (*S*_1_ *S*_2_)	–	0	–	–	–	–	–	–	–	–	–
Goldrich (*S*_1_ *S*_2_)	Katy (*S*_1_ *S*_2_)	‘G×K’	26	–	–	12	10	4	–	–	2∶1:1	2.92 (0.23)
Harcot (*S*_1_ *S*_4_)	Katy (*S*_1_ *S*_2_)	‘H×K’	44	–	–	20	–	4	7	13	2∶1:1∶2	3.68 (0.30)
Katy (*S*_1_ *S*_2_)	Canino (*S*_2_ *S*_C_)	‘K×C’	50	15	19	6	10	–	–	–	2∶2:1∶1	1.49 (0.69)
Canino (*S*_2_ *S*_C_)	Katy (*S*_1_ *S*_2_)	‘C×K’	88	32	15	29	12	–	–	–	2∶1:2∶1	0.74 (0.86)

Observed *S-RNase* genotypes, expected segregation ratios and χ^2^ values obtained for each population are indicated.

a *S*-genotypes for ‘Goldrich’, ‘Harcot’ and ‘Canino’ were prevously reported by Vilanova et al. (2005) and the *S*-genotype for ‘Katy’ was determined in this work.

b ‘K×K’ data correspond to three combined F_2_ populations obtained by self-pollinating ‘Katy’ in 2005, 2006 and 2010.

c Obtained seedlings.

d Expected ratios for a single mutation unlinked to the *S*-locus.

e Observed ratios do not differ significantly from expected at P<0.05 (barring ‘Katy’ self-pollination at P>0.01).

### SC in ‘Katy’ is Associated with a PPM Unlinked to the *S*-locus

To analyze the nature of SC in ‘Katy’, this cultivar was self-pollinated and reciprocally crossed with ‘Goldrich’, a self-incompatible cultivar sharing the same *S*-genotype. *S*-*RNase* genotyping of the progenies derived from the ‘Katy’ (*S*_1_ *S*_2_) self-pollination (Figure 1C) and the ‘Goldrich’ (*S*_1_ *S*_2_) × ‘Katy’ (*S*_1_ *S*_2_) outcross (Figure 1D) revealed three different *S*-genotypes (*S*_1_ *S*_1_:*S*_1_ *S*_2_:*S*_2_ *S*_2_) in both cases (Table 1). In turn, the ‘Katy’ (*S*_1_ *S*_2_) × ‘Goldrich’ (*S*_1_ *S*_2_) cross did not produce any seedling. Thus, ‘Katy’ pollen is able to grow through the ‘Goldrich’ pistil meanwhile ‘Goldrich’ pollen is rejected in the ‘Katy’ styles. According to these results, SI breakdown in ‘Katy’ may be due to a pollen-part mutation since ‘Katy’ is completely functional as a female parent. Indirect evidence supporting this hypothesis was also compiled from the *S*-genotype segregation ratio in ‘K×C’, because the number of *S*_2_ bearing genotypes is lower than that expected for a non-functional pistil-*S*_2_ determinant (Table 1). Moreover, both ‘Katy’ *S*-alleles are able to grow in ‘Goldrich’ and ‘Katy’ styles suggesting that the PPM is unlinked to the *S*-locus.

To complement these observations, we performed additional crosses with cultivars having different *S*-genotypes. Figure 1E shows the *S*-*RNase* genotyping of the ‘Harcot’ (*S*_1_ *S*_4_) × ‘Katy’ (*S*_1_ *S*_2_) population where *S*-genotypes fell into four classes (*S*_1_ *S*_1_:*S*_1_ *S*_2_:*S*_1_ *S*_4_:*S*_2_ *S*_4_) (Table 1). Two of these *S*-genotypes were unexpectedly obtained (*S*_1_ *S*_1_ and *S*_1_ *S*_4_) since pollen tubes carrying the *S*_1_-haplotype from ‘Katy’ were expected to be incompatible in ‘Harcot’ styles. On the other hand, reciprocal crosses with the cv. ‘Canino’ (*S*_2_ *S*_C_ *Mm*) produced four *S*-genotype classes (*S*_2_ *S*_C_:*S*_2_ *S*_2_:*S*_1_ *S*_C_:*S*_1_ *S*_2_). According to the two unlinked PPMs associated wtih SC in ‘Canino’ (*S*_C_ and *m*), these four *S*-genotypes were expected for the ‘K×C’ progeny (Table 1). Nevertheless, since pollen tubes having the *S*_2_-haplotype should be arrested in *S*_2_-styles, the *S*_2_ *S*_C_ and *S*_2_ *S*_2_ genotypes observed in the ‘C×K’ progeny were unexpected. The observed ratios for *S*-genotype segregations in ‘H×K’ and ‘C×K’ fit with that expected in a model where ‘Katy’ carries a heterozygous PPM affecting pollen-*S* function that is unlinked to the *S*-locus (2∶2:1∶1) with χ^2^ values of 3.68 and 0.74 (*P* = 0.30 and *P* = 0.86) (Tables 1 and 2). On the contrary, if we consider an heterozygous PPM linked in coupling to the incompatible *S*-allele or an homozygous PPM (linked or unlinked to the *S*-locus) the expected ratios (1∶1:1∶1) do not fit with the observed data with χ^2^ values of 13.6 and 13.5, respectively (*P*<0.004).

**Table 2:** Expected gamete and seedling genotypes formed from the outcross ‘Harcot’ (*S*_1_ *S*_4_) x ‘Katy’ (*S*_1_ *S*_2_) and the selfing of ‘Katy’ (*S*_1_ *S*_2_) considering ‘Katy’ heterozygous for a pollen-part mutation unlinked to the *S*-locus (*M’m’*).

Female gametes	Male gametes ‘Katy’ (*S*_1_ *S*_2_ *M’m’*)			
‘Harcot’ (*S*_1_ *S*_4_ *M’M’*)	*S*_1_ *M’* b	*S*_1_ *m’*	*S*_2_ *M’*	*S*_2_ *m’*
***S*** **_1_** ***M’***	X^a^	*S*_1_ *S*_1_ *M’m’*	*S*_1_ *S*_2_ *M’M’*	*S*_1_ *S*_2_ *M’m’*
***S*** **_4_** ***M’***	X	*S*_1_ *S*_4_ *M’m’*	*S*_2_ *S*_4_ *M’M’*	*S*_2_ *S*_4_ *M’m’*
‘Katy’ (*S*_1_ *S*_2_ *M’m’*)	***S*** **_1_** ***M’***	***S*** **_1_** ***m’***	***S*** **_2_** ***M’***	***S*** **_2_** ***m’*** b
***S*** **_1_** ***M’***	X	*S*_1_ *S*_1_ *M’m’*	X	*S*_1_ *S*_2_ *M’m’*
***S*** **_1_** ***m’***	X	*S*_1_ *S*_1_ *m’m’*	X	*S*_1_ *S*_2_ *m’m’*
***S*** **_2_** ***M’***	X	*S*_1_ *S*_2_ *M’m’*	X	*S*_2_ *S*_2_ *M’m’*
***S*** **_2_** ***m’***	X	*S*_1_ *S*_2_ *m’m’*	X	*S*_2_ *S*_2_ *m’m’*

a Pollen incompatibility.

b If *m’* was linked in coupling with *S*_2_ the *S*_2_ *M’* and *S*_1_ *m’* gametes from ‘Katy’ would not be formed, and conversely if *m’* was linked in coupling with *S*_1_ the *S*_1_ *M’* and *S*_2_ *m’* gametes would not be formed.

All performed crosses were shown to be compatible, barring ‘Katy × Goldrich’ cross, and fruit set ranged approximately from 15% (‘K×K’) to 34% (‘C×K’). Differences in germination rate and seedling fitness were striking. Only 59% of the ‘K×K’ inbred seeds produced healthy plants while this percentage increased to 82–96% in the outcrossed seeds.

### Molecular Analysis of the Self-compatible cv. ‘Katy’ (*S*_1_ *S*_2_)

To test whether the ‘Katy’ pollen tubes are not rejected in pistils bearing a matching *S*-allele as a consequence of SNPs or indels affecting *SFB*_1_ and *SFB*_2_, genomic DNA fragments containing both alleles were cloned and sequenced. Genomic sequences of *S*_1_ and *S*_2_-haplotype regions from the self-incompatible cv. Goldrich (*S*_1_ *S*_2_) were used as references [44]. No changes were found in the nucleotide sequences of the two cloned fragments (approximately 1.3 and 1.9 kb, respectively) containing the complete *SFB*_1_ and *SFB*_2_ open reading frames as well as their 5′ and 3′ adjacent flanking regions (∼110/390 and ∼70/470 bp from the 5′ and 3′ *SFB*_1_/*SFB*_2_ flanking regions, respectively).

**Figure 2:**
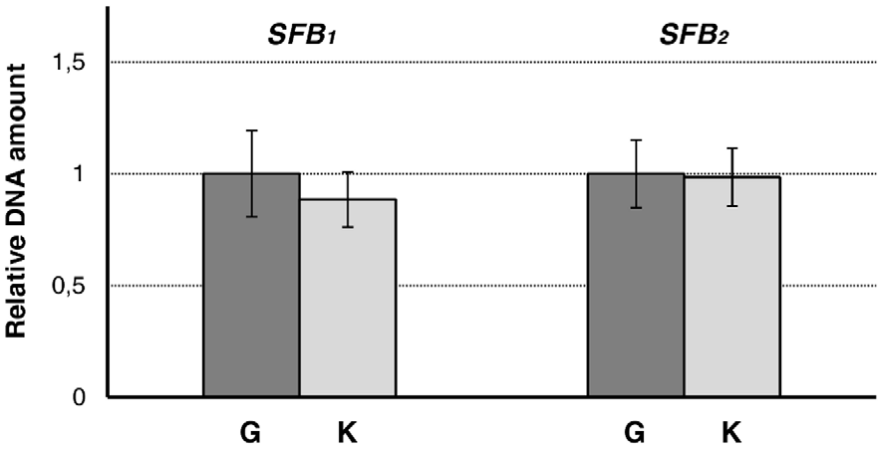
Relative DNA amount of *SFB*_1_ and *SFB*_2_ in ‘Goldrich’ (G) and ‘Katy’ (K). Quantities correspond to the average of two independent biological replicates repeated three times and were determined using *actin* as endogenous control. Bars indicate standard deviations.

PPMs identified in Solanaceae are mostly associated with *S*-allele duplications caused by polyploidy or induced mutations [45]. To discard this reason, we first examined the ploidy level in ‘Katy’ by flow cytometry analysis. The peaks of nuclei isolated from ‘Katy’ were coincident with those detected in the control diploid plant (‘Goldrich’), indicating that ‘Katy’ is a diploid (data not shown). A hypothetical duplication of the *SFB* alleles in ‘Katy’ was also tested by a real-time PCR-based gene dosage assay, but the relative DNA amounts detected for *SFB*_1_ and *SFB*_2_ were not significantly different between ‘Katy’ and the self-incompatible cv. ‘Goldrich’ (Figure 2).

**Figure 3:**
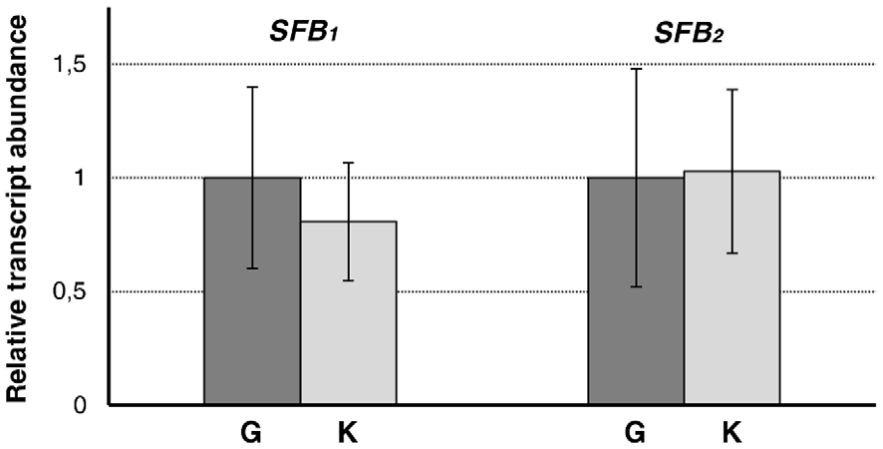
Relative transcript abundance of *SFB*_1_ and *SFB*_2_ in ‘Goldrich’ (G) and ‘Katy’ (K). Quantities correspond to the average of three independent biological replicates repeated three times. Bars indicate standard deviations.

Gene expression analysis showed that *SFB*_1_ and *SFB*_2_ alleles are specifically expressed in pollen in ‘Katy’ and ‘Goldrich’ (data not shown). Furthermore, relative transcript abundance of *SFB*_1_ and *SFB*_2_ in ‘Katy’ and ‘Goldrich’ was quantified by real-time RT-PCR using *actin* as endogenous control to normalize transcription values. No significant differences in the transcript levels were found for any of the two *SFB* alleles between ‘Katy’ and the self-incompatible cv. ‘Goldrich’ (Figure 3) discarding transcriptional repression of *SFB*s as the cause of SC.

### *S*-locus Unlinked PPM Conferring SC in ‘Katy’ is Located on Linkage Group 3

Overall, genetic and molecular evidence support a model where ‘Katy’ is heterozygous for a PPM unlinked to the *S*-locus that confers SC. The locus containing this PPM in ‘Katy’ was referred as *M’*-locus to distinguish it from the *M*-locus previously reported in ‘Canino’ [40]. Thus, according to the *S*- and *M’*-locus genotypes, ‘Katy’ was designated as *S*_1_ *S*_2_ *M’m’* (Table 2). Under the proposed genetic model, SSR markers linked to the *M’*-locus in ‘Katy’ selfing populations should be highly distorted, since only seedlings derived from ‘Katy’ pollen gametes carrying the *m’*-allele (*S*_1_ *m’* or *S*_2_ *m’*) could be obtained (Table 2). Thus, the expected ratio for a SSR marker segregating independently of the *M’*-locus in the F_2_ populations is 1∶2:1 while that for an absolutely linked SSR is 1∶1. On this assumption, genome-wide distributed SSR markers were tested to look for associations with the *M’*-locus. Thereby, 118 SSR markers distributed across the eight *Prunus* chromosomes (ranging from 9 in LG7 to 34 in LG3) were selected for mapping (Tables S1 and S2). Fifty-five of these SSRs (47%) were found to be polymorphic in ‘Katy’ and, subsequently, tested in the ‘K×K_05_’ and ‘K×K_06_’ progenies (Table 3). According to the genetic maps constructed for each group, the maximum genetic distance estimated between any pair of markers was ∼52 cM in LG5 (Table 3). In terms of the physical distance, determined from the peach genome sequence, the major gap was found in LG1 (∼23 Mb). Considering the estimated sizes for the peach genome (∼290 Mb) and for the *Prunus* general map (519 cM) [46], the relationship between physical and genetic distances is ∼0.56 Mb/cM on average. Accordingly, the LG1 23 Mb gap should correspond to <45 cM. Consequently, in the most unfavourable scenario, distance to *M’*-locus should be lower than 25 cM and recombination frequency lower than 0.25. In this hypothetical case, the expected ratio for a SSR linked to the *M’*-locus would be 1∶4:3, and only markers located on LG3 and LG6 fulfill this prediction and show skewed segregations (χ^2^>5.99 with *P*<0.05 for 2 d.f.) (Table 3).

**Table 3:** Identification of segregation distortion SSR loci distributed throughout the eight linkage groups (LG) of ‘Katy’ using the ‘K×K_05_’ and ‘K×K_06_’ populations.

LG	Locus	Peach Mb^a^	Apricot cM^b^	Seg. type^c^	A	H	B	Total	?^2^ (*P*-value)^d^
1	Gol051	4,69	00,0 (0,26)	<abxab>	12	22	12	46	0,09 (0,96)
1	EPPCU0027	9,51	30,7 (0,00)	<abxab>	17	19	9	45	3,93 (0,14)
1	pchcms4	9,51	30,7 (0,36)	<abxab>	18	19	9	46	4,91 (0,09)
1	CPPCT045	32,02	77,5	<abxab>	7	30	9	46	4,44 (0,11)
2	ssrPaCITA19	13,01	00,0 (0,17)	<abxab>	18	18	10	46	4,96 (0,08)
2	UDP98-411	20,17	17,2 (0,18)	<abxab>	13	24	9	46	0,78 (0,67)
2	CPSCT021	23,74	36,9 (0,03)	<abxab>	10	27	9	46	1,44 (0,49)
2	CPSCT031	25,15	40,3	<abxab>	10	26	10	46	0,78 (0,68)
3	ssrPaCITA23	02,70	00,0 (0,17)	<abxab>	8	25	13	46	1,44 (0,49)
3	UDAp468	04,85	18,0 (0,24)	<abxab>	20	16	9	45	9,13 (0,01)^e^
3	PGS3_03	16,41	44,7 (0,23)	<abxab>	5	20	21	46	11,91 (0,003)^e^
3	EPPCU7190	19,78	69,0	<abxab>	18	25	2	45	12,29 (0,002)^e^
4	UDP96-003	08,76	00,0 (0,12)	<abxab>	9	25	12	46	0,74 (0,69)
4	BPPCT040	06,46	12,0 (0,13)	<abxab>	10	27	9	46	1,44 (0,49)
4	UDAp404	–	25,4	<abxab>	12	26	8	46	1,48 (0,48)
5	PGS5_02	00,48	00,0 (0,39)	<abxab>	8	24	12	44	1,09 (0,58)
5	UDAp452	13,76	52,3 (0,35)	<abxab>	8	23	15	46	2,13 (0,34)
5	CPSCT006	11,53	95,1	<abxab>	10	25	11	46	0,39 (0,82)
6	PGS6_04	04,95	00,0 (0,20)	<abxab>	5	23	16	44	5,59 (0,06)
6	UDAp420	08,14	21,6 (0,10)	<abxab>	6	20	20	46	9,30 (0,01)^e^
6	UDAp489	16,82	31,9 (0,09)	<abxab>	18	21	7	46	5,61 (0,06)
6	Ma027a	20,90	41,3 (0,23)	<abxab>	16	25	4	45	6,96 (0,03)^e^
6	ssrPaCITA12	27,84	64,3 (0,03)	<abxab>	7	22	17	46	4,44 (0,11)
6	Locus-*S*	26,45	67,6	<abxab>	6	23	17	46	5,26 (0,07)
7	CPSCT026	10,98	00,0 (0,00)	<abxab>	13	23	10	46	0,39 (0,82)
7	CPPCT022	10,23	00,0 (0,26)	<abxab>	13	23	10	46	0,39 (0,82)
7	CPSCT042	17,08	29,2	<abxab>	10	20	16	46	2,35 (0,31)
8	PGS8_02	03,28	00,0 (0,03)	<abxab>	7	24	7	38	2,63 (0,27)
8	PGS8_05	07,39	03,4 (0,04)	<abxab>	8	25	11	44	1,23 (0,54)
8	UDAp401	10,50	07,2 (0,00)	<abxab>	10	23	12	45	0,20 (0,90)
8	UDAp470	12,61	07,2 (0,05)	<abxab>	10	24	12	46	0,26 (0,88)
8	M6a	15,03	11,8	<abxab>	9	25	11	45	0,73 (0,69)

a Marker position (Mb) within the corresponding peach genome scaffolds which sizes were estimated by IPGI (scaffold_1, 46.88 Mb; _2, 26.81 Mb; _3, 22.02 Mb; _4, 30.53 Mb; _5, 18.50 Mb; _6, 28.90 Mb; _7, 22.79 Mb and _8, 21.83 Mb).

b Map position (cM) and rec. frequencies (in brackets) estimated by JoinMap 3.0.

c Segregation type as per JoinMap 3.0.

d Chi-square test was performed for the expected ratio 1∶2:1 (<abxab>).

e Observed ratios differ significantly from expected at *P*<0.05 for 2 degrees of freedom.

In agreement with the segregation of the *S*-genotypes in the analyzed populations, the *M’*-locus is proposed to be unlinked to the *S*-locus (Table 1). Therefore, LG3 or a region far from the LG6 distal end, where the *S*-locus is located, are likely positions for the *M’*-locus. To discern between these two possibilities, a more detailed SDL analysis was performed in LG3 (Table 4) and LG6 (Table S1) by including the ‘K×K_10_’ population and additional markers. On one side, this analysis showed that LG6 distorted markers are partially linked to the *S*-locus (i.e. Ma027a shows a recombination frequency of 0.26 at LOD 3.3 with the *S*-locus). On the other, the magnitude of the segregation distortion detected in LG6 (χ^2^ = 15.28 with *P* = 5×10^−4^ for PGS6_07) lower than that found in LG3 (χ^2^ = 31.30 with *P* = 1.6×10^−7^ for PGS3_23). This is due to the lower imbalance between homozygous genotypes found in PGS6_07 (7B against 32A) when compared with PGS3_23 (0B against 37A) (Table 4 and Table S1). It is inferred from the model that pollen gametes carrying SSR alleles linked in repulsion phase with the PPM would not grow into incompatible styles. Therefore, homozygous genotypes for these SSR alleles should not be obtained in the progeny, as observed for the LG3 SSR distorted markers and particularly for PGS3_23 (Table 4). Thus, both arguments support LG3 as the most likely location for the *M*’-locus allowing us to discard LG6.

**Table 4:** Identification of segregation distortion SSR loci distributed throughout the ‘Katy’ LG3 using data from the ‘K×K’ F_2_ population.

Locus	Peach Mb^a^	Apricot cM^b^	Seg. type^c^	A	H	B	Total	?^2^ (*P*-value)^d^
MA066a	02,40	00,0 (0,03)	<abxab>	15	46	25	86	2,74 (0,25)
ssrPaCITA23	02,70	02,3 (0,10)	<abxab>	16	44	27	87	2,79 (0,25)
UDAp468	04,85	12,1 (0,08)	<abxab>	16	38	31	85	6,25 (0,04)^e^
BPPCT039	05,80	19,6 (0,30)	<abxab>	13	42	30	85	6,81 (0,03)^e^
PGS3_03	16,41	39,2 (0,07)	<abxab>	4	46	35	85	23,19 (9×10^−6^)^e^
PGS3_12	17,38	46,3 (0,01)	<abxab>	4	44	35	83	23,46 (8×10^−6^)^e^
PGS3_15	17,71	46,9 (0,03)	<abxab>	4	45	32	81	20,36 (4×10^−5^)^e^
PGS3_22	18,49	49,2 (0,03)	<abxab>	3	45	35	83	25,27 (3×10^−6^)^e^
PGS3_23	18,61	51,1 (0,05)	<abxab>	0	48	37	85	33,64 (5e-8)^e^
PGS3_28	19,14	55,1 (0,02)	<abxab>	3	49	31	83	21,60 (2×10^−5^)^e^
PGS3_32	19,60	56,8 (0,00)	<abxab>	4	48	31	83	19,60 (6×10^−5^)^e^
PGS3_33	19,66	56,9 (0,03)	<abxab>	4	50	30	84	19,14 (7×10^−5^)^e^
AMPA119	20,00	59,0 (0,00)	<abxab>	4	47	35	86	23,09 (9×10^−6^)^e^
EPPCU7190	19,78	59,1 (0,10)	<abxab>	4	47	33	84	21,21 (2×10^−5^)^e^
CPDCT027	21,67	67,1 (0,12)	<abxab>	9	40	32	81	13,07 (0,001)^e^
EPPCU0532	22,00	72,0	<abxab>	12	42	21	75	3,24 (0,20)

a Marker position (Mb) within the peach genome scaffold_3 which size estimated by IPGI was 22.02 Mb.

b Map position (cM) and rec. frequencies (in brackets) estimated by JoinMap 3.0.

c Segregation type as per JoinMap 3.0.

d Chi-square test was performed for the expected ratios 1∶2:1 (<abxab>) (a) and 1∶1 (<nn×np>/<ef×eg>/<ab×cd>) (b).

e Observed ratios differ significantly from expected at *P*<0.05 for 2 (a) or 1 degrees of freedom (b).

### High-density Mapping of the *M’*-locus on chr.3

To construct a high-density map of the *M’*-locus region on chr.3, 102 SSRs identified from the peach scaffold_3 sequence by Zuriaga *et al.* [40] (Table S2) and 18 additional SSRs available from the GDR website [47] were tested in ‘Katy’. A higher percentage of these SSRs did not amplify or produced multi-band patterns in ‘Katy’ (40%) when compared with both ‘Goldrich’ and ‘Canino’ (∼30%). However, polymorphism of amplified SSRs was similar between ‘Goldrich’ and ‘Katy’ (∼55%) and significantly higher than that found in ‘Canino’ (23%) (Table S2). Polymorphic SSRs in ‘Katy’ were tested in 87 trees from the ‘K×K’ F_2_ population. Sixteen of them were mapped, forming a LG3 genetic map of 72 cM with an average marker density of 0.22 marker/cM (Table 4). This marker density increased up to 0.62 marker/cM in the region flanked by the most distorted markers PGS3_12 and AMPA119 (Table 4). An additional LG3 map obtained from the outcrossing population ‘C×K’ was found to be essentially collinear with the ‘K×K’ map (sharing >80% markers), except for a single order change between AMPA119 and PGS3_32 (data not shown). The SDL associated with the *M’*-locus region were confirmed by analyzing 60 additional seedlings derived from the outcrosses ‘H×K’, ‘G×K’ and ‘C×K’ for all sixteen LG3 markers (Table 5). These seedlings were selected by their *S*-genotypes, so that they could only be derived from the fertilization with a ‘Katy’ pollen gamete carrying the PPM (*m*’) and, therefore, directly assigned to the *M’ m’* genotype (Table 2). Skewed segregations in selfing (F_2_) and outcrossing populations suggested that the *M’*-locus is roughly located between PGS3_22 and PGS3_28 (Tables 4 and 5).

**Table 5:** Identification of segregation distortion SSR loci distributed throughout the ‘Katy’ LG3, using data from subsets of the outcrossing populations ‘H×K’, ‘G×K’ and ‘C×K’ carrying the PPM.

Locus	PeachMb^a^	Population^b^	Seg.Type^c^	-c	-d	-e	-g	-n	-p	Total	?^2^ (*P*-value)^d^
MA066a	02,40	H×K−/G×K	<efxeg>/<nnxnp>			5	6	11	14	36	0,44 (0,50)
ssrPaCITA23	02,70	H×K/G×K	<efxeg>			16	20			36	0,44 (0,50)
UDAp468	04,85	H×K/C×K	<efxeg>			21	14			35	1,40 (0,24)
BPPCT039	05,80	H×K/C×K	<abxcd>/<efxeg>	3	8	13	9			33	2,46 (0,12)
PGS3_03	16,41	H×K/C×K	<efxeg>			33	2			35	27,46 (1.6e−7)^e^
PGS3_12	17,38	All three	<efxeg>/<nnxnp>			23	1	34	2	60	48,60 (0,00)^e^
PGS3_15	17,71	C×K	<efxeg>			24	0			24	24,00 (9.6e−7)
PGS3_22	18,49	All three	<efxeg>/<nnxnp>			36	0	24	0	60	60,00 (0,00)^e^
PGS3_23	18,61	All three	<efxeg>/<nnxnp>			36	0	24	0	60	60,00 (0,00)^e^
PGS3_28	19,14	All three	<nnxnp>					60	0	60	60,00 (0,00)^e^
PGS3_32	19,60	All three	<efxeg>/<nnxnp>			11	0	48	1	60	56,07 (0,00)^e^
PGS3_33	19,66	All three	<abxcd>/<efxeg>	0	11	48	1			60	56,07 (0,00)^e^
AMPA119	20,00	All three	<efxeg>			59	1			60	56,07 (0,00)^e^
EPPCU7190	19,78	All three	<efxeg>			59	1			60	56,07 (0,00)^e^
CPDCT027	21,67	All three	<abxcd>/<nnxnp>	31	3			19	5	58	30,41 (3e−8)^e^
EPPCU0532	22,00	H×K/G×K	<efxeg>/<nnxnp>			11	0	21	3	35	24,03 (9.5e−7)^e^

a Marker position (Mb) within the peach genome scaffold_3 which size estimated by IPGI was 22.02 Mb.

b *S*-genotypes of the selected seedlings were: *S*_1_ *S*_1_ and *S*_1_ *S*_4_ in ‘H×K’; *S*_2_ *S*_2_ and *S*_C_ *S*_2_ in ‘C×K’; *S*_1_ *S*_1_, *S*_1_ *S*_2_ and *S*_2_ *S*_2_ in ‘G×K’.

c Segregation type as per JoinMap 3.0.

d Chi-square test was performed for the expected ratios 1∶2:1 (<abxab>) (a) and 1∶1 (<nn×np>/<ef×eg>/<ab×cd>) (b).

e Observed ratios differ significantly from expected at *P*<0.05 for 2 (a) or 1 degrees of freedom (b).

**Figure 4:**
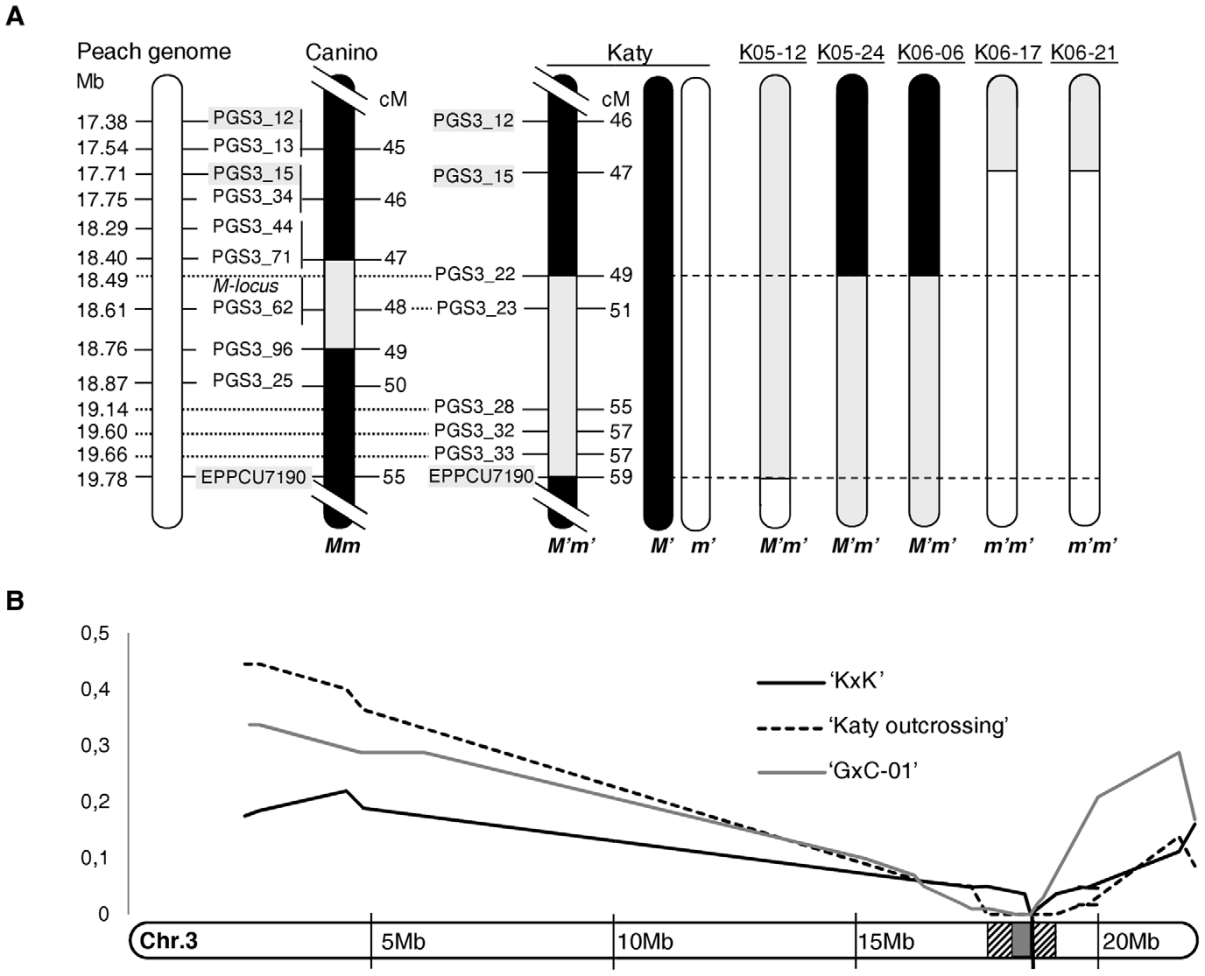
Mapping of the *M’*-locus and macro-synteny within *Prunus*. (a) Graphical LG3 maps of K×K-recombinant hybrids at the *M’*-locus. The corresponding map region between markers PGS3_12 and EPPCU7190 is shown for ‘Katy’ and ‘Canino’. Distances in centimorgan (cM) are shown on the right of the apricot maps and their corresponding positions in megabases (Mb) on the peach genome are shown on the left. *Black vertical* bars represent self-incompatible (*M’M’*) chromosomal regions, while *grey* (*M’m’*) and *white* bars (*m’m’*) correspond to self-compatible chromosomal regions. Recombinant seedlings are numbered at the top. (b) Predicted positions for the *M*- and *M’*-loci on the peach chr.3 according to the relative frequency of individuals lacking SSR alleles in coupling phase with the PPM (*Y-axis*). The *black line* represents data corresponding to the ‘K×K’, the *dashed line* to ‘Katy’ outcrossing populations (‘H×K’, ‘G×K’ and ‘C×K’) and the *grey line* to the ‘G×C-01’ population.

To define the *M’*-locus location more consistently, not only considering distortions but also on the basis of genotyping data, an additional mapping strategy was performed. As described above, all ‘K×K-F_2_’ trees could only be derived from pollen gametes with genotype *S*_1_ *m’* or *S*_2_ *m’*, having either the *M’m’* or the *m’m’* genotype. To discriminate between these two genotypes the screening of F_3_ offsprings was necessary. Thereby, twelve ‘K×K-F_2_’ individuals, with recombination breakpoints mapping to the LG3 region between UDAp468 and CPDCT027, were self-pollinated to obtain F_3_ populations. Six of them (K05-15, K05-21, K06-18, K06-25, K06-34 and K06-37) were finally discarded for the analysis due to the low number of embryos obtained (less than 7 in four cases) or because they were redundantly represented (other F_2_ individuals with larger F_3_ populations have identical SSR genotypes in this genomic region). The six F_3_ populations obtained from the remaining F_2_ recombinants (K05-12, K05-24; K06-05, K06-06, K06-17 and K06-21) were tested for a subset of 6 SSRs encompassing the *M’*-locus (PGS3_13/PGS3_32 interval) (Table 6). Those SSR markers heterozygous in the F_2_ recombinant (H) were expected to segregate 1∶1 in the F_3_ population when the F_2_ recombinant had the *M’m’* genotype and 1∶2:1 if it had the *m’m’* genotype (Table 6). According to the segregation of these markers (A, H or B as per JoinMap 3.0 notation) the *M’*-locus was proposed to be flanked by PGS3_22 and EPPCU7190 markers within an interval of 9.4 cM. Graphical ordering of genotype data enable the positioning of recombination breakpoints to confirm map order (Figure 4A).

**Table 6:** *M’*-locus genotyping of trees belonging to the ‘K×K_05_’ and ‘K×K_06_’ F_2_ populations.

SSR genotypes of F_3_ progenies from ‘K×K_05_’ and ‘K×K_06_’ F_2_ trees													
K05-12	Gen^a^	A	H	B	?^2^ (P-value)	*M’*-locus	K06-05	Gen	A	H	B	?^2^ (P-value)	*M’*-locus
PGS3_12	H						PGS3_12	H					
PGS3_13							PGS3_13						
PGS3_15							PGS3_15						
PGS3_22	H	0	14	15	0,03 (0,85)	*M’ m’*	PGS3_22	H	0	12	6	2,00 (0,16)	*M’ m’*
PGS3_23							PGS3_23						
PGS3_28							PGS3_28						
PGS3_32							PGS3_32						
EPPCU7190	B						EPPCU7190	H					
**K05-24**	**Gen**	**A**	**H**	**B**	**χ^2^ (P-value)**	***M’*** **-locus**	**K06-06**	**Gen**	**A**	**H**	**B**	**χ^2^ (P-value)**	***M’*** **-locus**
PGS3_12	A						PGS3_12	A					
PGS3_13							PGS3_13						
PGS3_15	A	63	0	0			PGS3_15	A	24	0	0		
PGS3_22							PGS3_22						
PGS3_23							PGS3_23						
PGS3_28	H	0	31	32	0,02 (0,90)	*M’ m’*	PGS3_28	H	0	9	15	1,50 (0,22)	*M’ m’*
PGS3_32							PGS3_32						
EPPCU7190	H						EPPCU7190	H					
**K06-17**	**Gen**	**A**	**H**	**B**	**χ^2^ (P-value)**	***M’*** **-locus**	**K06-21**	**Gen**	**A**	**H**	**B**	**χ^2^ (P-value)**	***M’*** **-locus**
PGS3_12	H						PGS3_12	H					
PGS3_13							PGS3_13						
PGS3_15	H	10	8	3	5,85 (0,05)	*m’ m’*	PGS3_15	H	5	15	9	1,14 (0,57)	*m’ m’*
PGS3_22							PGS3_22						
PGS3_23	B	0	0	21			PGS3_23	B	0	0	29		
PGS3_28							PGS3_28						
PGS3_32							PGS3_32						
EPPCU7190	B						EPPCU7190	B					

*M’*-genotypes were determined by PCR-based amplification of SSR markers (PGS3_13, PGS3_15, PGS3_22, PGS3_23, PGS3_28 and PGS3_32) in the F_3_ progenies. Number of embryos falling into each genotypic class (A, H or B) are indicated and *bold lines* represent recombination breakpoints.

a ‘Gen’ indicates the SSR genotype for each F_2_ recombinant.

b Chi-square χ^2^ and *P* values for the expected segregation ratios 1∶2:1 (*m’m’*) and 1∶1 (*M’m’*) obtained from each independent F_3_ population.

### Macro- and Microsynteny Analysis of the *M’*-locus in Apricot

Eleven out of the sixteen SSR markers contained in the ‘Katy’ LG3 map had been previously mapped in the ‘Canino’ LG3 [40]. As a whole, these markers were found to be collinear between both maps (8 out of 11) but some order changes regarding PGS3_33, AMPA119 and EPPCU0532 were observed at the distal chromosome end (data not shown). In turn, marker order in the ‘Katy’ LG3 map was completely collinear with the physical position of the markers in the peach genome (Table 4 and Figure 4). Unfortunately, most of the markers surrounding the *M*-locus in ‘Canino’ LG3 were found to be monomorphic in ‘Katy’ and therefore could not be mapped (Table S3). Genetic differences between ‘Katy’ and ‘Canino’ were detected across the whole genome, they share only 38,8% of their SSR alleles and show a Neís genetic distance of 0,83 (Table S4). Indeed, only a few collinear markers, such as PGS3_12, PGS3_15 and EEPCU7190, were useful to define a syntenic region between both apricot maps containing the *M*- and *M*’-loci and corresponding to a physical interval between 17.38–19.78 Mb in the peach genome (Figure 4A). The PGS3_22/EEPCU7190 interval comprising the *M*’-locus in ‘Katy’ corresponds to ∼1.29 Mb in the peach syntenic genomic region (between 18.490–19.780 Mb positions). Meanwhile, in ‘Canino’ the *M*-locus was predicted to be flanked by PGS3_71 and PGS3_96 markers within an interval of 1.8 cM corresponding to ∼364 Kb in the peach genome (between 18.399–18.763 Mb positions) [40]. Therefore, there is an overlapping interval between these two regions spanning ∼273 kb. To have a complementary view of the predicted positions for the *M*- and *M’*-loci, the relative frequency of individuals lacking SSR alleles in coupling phase with the PPM (expected to be zero in those markers absolutely linked) was represented graphically on the peach chr.3 (Figure 4B). To do this, only individuals carrying the ‘Canino’ *m* mutated allele from the ‘G×C-01′ population [40] or the *m’* allele from ‘K×K’ and ‘Katy’ outcrossing populations were computed. This analysis showed frequency values of zero in shorter overlapping intervals: PGS3_23 (18.61 Mb) in ‘K×K’, PGS3_22/PGS3_28 (18.49–19.14 Mb, ∼650 Kb) in ‘Katy’ outcrosses and PGS3_44/PGS3_62 (18.29–18.61 Mb, ∼320 Kb) in ‘G×C–01’.

The genomic landscape of the ∼1.29 Mb peach region syntenic to the apricot *M’*-locus contains 223 predicted gene transcripts as annotated by IPGI. Forty-two of these transcripts (located in the overlapping interval) were shared in common with the ‘Canino’ *M*-locus. BLASTP analysis of the ORFs against The Arabidopsis Information Resource (TAIR) database, with an exp. value cut-off <1e^−6^, was used by IPGI to predict gene functions based on homology to *Arabidopsis*. Table S5 includes the results of the BLASTP analysis for the ORFs comprised in the *M’*-locus region (IPGI) and indicates those *Prunus*/*Arabidopsis* gene pairs that are best-reciprocal BLASTP hits identifying putative orthologues. According to the large-scale gene expression analysis performed by Wang *et al.* [48] in *Arabidopsis* mature pollen, hydrated pollen and pollen tubes using Affymetrix ATH1 Genome Arrays, up to 53 of these *Arabidopsis* homologues were found to be pollen-expressed (Table S5).

## Discussion

### Loss of Function of an *S*-locus External Factor is Responsible for SI Breakdown in ‘Katy’ (*S*_1_ *S*_2_)

In this work the North-American apricot cv. ‘Katy’, released by Zaigeŕs Genetics (Modesto, CA, USA) in 1978 [41], was confirmed as self-fruitful and its *S*-genotype was determined as *S*_1_ *S*_2_ following the nomenclature established by Burgos *et al.* [42]. However, previous reports assigned to ‘Katy’ the *S*-genotypes *S*_8_ *S*_C_ [49] and *S*_1_ *S*_8_ [50]. In addition, these two manuscripts referred ‘Katy’ as a spontaneous cultivar native to Europe and lately introduced to China. Therefore, both the *S*-genotype and the geographic origin proposed by these authors suggest that the cultivars they analyzed might be different from the cv. ‘Katy’ we describe here. Wu *et al*. [50] also suggest that SC in ‘Katy’ is associated with PPMs that, according to the segregation of *S*-genotypes, seem to exert a polygenic control. Again, this is not the case in the Zaigeŕs ‘Katy’ where SC is associated with a single PPM, however a sort of kinship between the two cultivars can not be discarded.

To investigate the genetics of SC, ‘Katy’ (*S*_1_ *S*_2_) was self-pollinated and reciprocally crossed with the self-incompatible cv. ‘Goldrich’ (*S*_1_ *S*_2_) [51], [52]. ‘Katy’ pollen tubes bearing either the *S*_1_- or the *S*_2_-haplotype were able to grow in ‘Katy’ and ‘Goldrich’ pistils and to complete fertilization, producing the three *S*-genotype classes expected for an F_2_ population (*S*_1_ *S*_1_:*S*_1_ *S*_2_:*S*_2_ *S*_2_). However, no progeny was obtained in the reciprocal cross using ‘Katy’ as female parent. These results would support a PPM unlinked to the *S*-locus as the cause for SC. Crosses performed with other cvs. such as ‘Harcot’ (*S*_1_ *S*_4_) and ‘Canino’ (*S*_2_ *S*_C_) reinforce this conclusion, since seedlings carrying the ‘Katy’ *S*_1_- (when crossing with ‘Harcot’) and the *S*_2_-haplotype (when crossing with ‘Canino’) were also obtained. Moreover, segregation ratios in all performed crosses fit with a model where ‘Katy’ is heterozygous for the PPM conferring SC (*M’m’*) (see Table 2).

Interestingly, in the ‘K×K’ and ‘G×K’ populations the number of seedlings homozygous for the *S*_1_-haplotype (20) is significantly lower than that for the *S*_2_-haplotype (43) (see Table 1). Similar deviations were observed by Wünsch and Hormaza [26] when the sweet cherry cv. ‘Cristobalina’ was self-pollinated. Following their reasoning, several causes might explain these deviations such as postzygotic selection against homozygous embryos, linkage in coupling between the mutated allele of the modifier factor (*m*) and the *S*_2_-allele or differences in the pollen competitive capacity to grow through the style (depending on the *S*-haplotype). In this particular case, a hypothetical effect of postzygotic selection would explain the reduced number of *S*_1_ *S*_1_ but not the high number of *S*_2_ *S*_2_ genotypes. Regarding the second reason, neither the segregation ratios observed in different populations nor the SDL analysis support a linkage between the *M’*-and the *S*-locus. Therefore, a lower growth capacity for pollen gametes bearing the *S*_1_-haplotype is regarded as the most acceptable hypothesis to explain this discrepancy.

SC caused by loss of pollen-*S* function has been usually found to be associated with mutations (mainly indels) of the *SFB* genes in different *Prunus* species such as sweet cherry [15]–[17], apricot [18], Japanese apricot [15], peach [14] and sour cherry [19]. However, sequence analysis revealed no mutations or indels affecting any of the two ‘Katy’ *SFB* alleles discarding this as the cause of SI breakdown. In Solanaceae, self-compatible PPMs may arise from *S*-allele duplications located in a centric fragment, in a non-S chromosome or linked to the *S*-locus leading to the formation of *S*-heteroallelic pollen [45]. According to the segregations obtained in the performed crosses, *S*-allele duplications did not seem probable in ‘Katy’ (all descendants should have had the *S*_1_ *S*_2_ genotype), even so, we discarded that possibility showing that *SFB* gene dosage is equivalent between ‘Katy’ and the self-incompatible cv. ‘Goldrich’. *S*-allele duplications may also result from polyploidy but ‘Katy’ was confirmed as diploid by flow cytometry analysis and by marker segregation and mapping in all crosses. These results rule out competitive interaction resulting from *S*-heteroallelic pollen as the cause of SC in ‘Katy’.

Altogether, it can be hypothesized that the loss-of-function of a *S*-locus unlinked factor gametophytically expressed in pollen causes breakdown of SI in ‘Katy’. Moreover, according to the relative abundance of *SFB*_1_ and *SFB*_2_ transcripts in ‘Katy’, when compared with the reference cv. ‘Goldrich’, the hypothetical defective factor in ‘Katy’ does not seem to affect their expression. These characteristics of the self-compatible mutant ‘Katy’ resemble those of other self-compatible pollen-part mutants defective for non *S*-locus factors already found in *Prunus*. For instance, gene duplications and modified transcription levels of the *S*-locus genes were also discarded as the cause of SC in the *Prunus avium* cv. ‘Cristobalina’ [53] and the *Prunus armeniaca* cv. ‘Canino’ [18]. According to the classification established by McClure *et al.* [30] the modifier factor in ‘Katy’ would belong to the group of modifier genes required for pollen rejection but with no wider role in pollination. Although no direct evidence is available about its possible function, last findings in *Prunus* may provide some clue in this respect. For instance, the PavSSK1 and PavCul1 proteins recently identified by Matsumoto *et al.* [39] in *Prunus avium* are proposed to form the SCFSFB E3 ubiquitine ligase complex involved in *S*-RNases degradation. Therefore, the loss-of-function of any of them would predictably lead to SC. However, none of these two genes is located in LG3 where the *M*’-locus region is found and so they can be discarded as a possible cause of SC in ‘Katy’. On the other hand, Tao and Iezzoni [24] proposed an alternative model for the GSI in *Prunus* where a *S*-RNase inhibitor would be the target for the SCFSFB ubiquitination complex instead of the *S*-RNases. If the modifier factor found in ‘Katy’ was this hypothetical inhibitor, its loss-of-function would lead to SI and not to SC what also rules out this possibility. Further research will therefore be necessary to reveal the SI related function affected by the PPM in ‘Katy’.

### PPMs Conferring SC in ‘Katy’ and ‘Canino’ Apricots are both Located at the chr. 3 Distal End

To facilitate future identification and cloning, the ‘Katy’ GSI mutated modifier gene locus (*M’*-locus) was mapped following a two-steps strategy. First, we hypothesized that those markers linked with the *M’*-locus should be highly distorted in the populations obtained from crosses where ‘Katy’ was the pollen parent, since only ‘Katy’ pollen tubes carrying the *m’*-allele would be able to grow. In other words, the *M’*-locus genomic region should correspond to a segregation distortion locus (SDL), a chromosomal region that causes distorted segregation ratios [54] To identify this kind of regions, ‘K×K_05_’ and ‘K×K_06_’ populations, which all trees carry the PPM, were tested for genome-wide distributed SSRs to detect SDL by examining changes in genotypic frequencies. Attending to segregation of pollen alleles, two SDL were found in LG3 and LG6 but a deeper analysis showed that LG6 markers were partially linked to the *S*-locus and only moderately distorted. Consequently, LG3 was predicted as the most likely location for the *M’*-locus. Distortion in LG6 seems more plausibly related to the different capacity of *S*_1_ and *S*_2_-pollen gametes for growing through the style. Further analyses are in progress to confirm this point.

In a second step, to refine *M’*-locus mapping, chr.3 specific SSRs were analyzed to estimate their segregation distortion ratios in selfing (F_2_) and outcrossing populations obtained by using ‘Katy’ as pollen parent. Additionally, indirect *M’*-locus genotyping was performed by analyzing linked SSRs in the F_3_ offspring of six selected ‘K×K’ F_2_ trees. Recombination breakpoints in five of these trees defined a 9.4 cM interval for the ‘Katy’ *M’*-locus that corresponds to ∼1.29 Mb in the peach genome (18.49–19.78 Mb) and overlaps ∼273 Kb with that established for the *M*-locus in ‘Canino’ [40]. A non *S*-locus PPM conferring SC to the *P. avium* cv. ‘Cristobalina’ was also mapped on the LG3 by Cachi and Wünsch [55]. However, it was tentatively predicted to be downstream the EMPaS02 marker (∼20,0 Mb) and therefore, if confirmed, the position for this locus is not coincident with those for the *M-* and *M’*-loci in apricot. Different map locations for PPMs would support different defective genes as responsible for SC in sweet cherry and apricot, but this point still requires confirmation. Particularly in apricot, SSR markers showing the highest distortion values associated with the PPMs in ‘Canino’ (PGS3_62) and ‘Katy’ (PGS3_23) are located in very close positions (18.612 and 18.608 Mb, respectively). Thus, in the light of the similarities found between the apricot cvs. ‘Katy’ and ‘Canino’ (i.e. genetics of SC, *M*- and *M’*-locus mapping positions, etc.) it is tempting to speculate that both PPMs causing SC might be affecting the same gene, however no conclusive evidence is yet available on this point. Only 42 genes are shared in common between *M*- and *M’*-locus [40] and, if this was the case, the availability of two different PPMs would be very helpful to identify the modifier gene. Interestingly, both cultivars have different geographic origins (i.e. ‘Katy’ is a North-American apricot selection [41] and ‘Canino’ is a local Spanish apricot [18]) and, according to the analysis of gemome-wide distributed SSRs, they seem to be genetically unrelated. This prompt us to speculate that both PPMs (being or not the same) may have arisen independently.

According to the peach syntenic genome region annotated by IPGI, the apricot *M’*-locus is predicted to contain about 223 gene transcripts. Based on sequence similarity, putative *Arabidopsis* orthologues were suggested for many of these *Prunus* genes [56] and, according to Movahedi *et al.* [57], a consistent tissue-specific expression might be expected for the reported gene pairs. Under this general rule, a high number of genes scattered throughout the *M’*-locus (up to 53) might be pollen-expressed fulfilling one of the main requirements for the SI ‘Katy’ modifier gene. Nevertheless, those genes whose orthologues are not pollen-expressed should not be discarded because inferred orthologues do not always have the same biological function [57]. Gene function annotation might also be helpful to select candidate genes for the SI ‘Katy’ modifier gene. Unfortunately, the hypothetical roles suggested for this factor are still merely speculative hindering this approach. In view of the limitations for these strategies and considering the high number of ORFs comprised within the *M’*-locus, narrowing down the mapping region will be an essential step to identify the SI modifier gene in ‘Katy’. In summary, ‘Katy’ does not only provide an additional *S*-locus unlinked source of SC, a desired trait for apricot breeding programs, but also becomes a very useful tool to dissect the molecular genetics behind pollen-pistil interactions in *Prunus*.

## Materials and Methods

### Plant Material

Four apricot cvs. ‘Goldrich’, ‘Canino’, ‘Harcot’ and ‘Katy’, the progenies derived from the outcrosses ‘Goldrich × Katy - 2005’ (‘G×K’), ‘Canino × Katy - 2007’ (‘C×K’), ‘Katy × Canino - 2007’ (‘K×C’) and ‘Harcot × Katy - 2005’ (‘H×K’), and the F_2_ populations obtained by selfing ‘Katy’ in 2005 (‘K×K_05_’) (*N* = 16), 2006 (‘K×K_06_’) (*N* = 37) and 2010 (‘K×K_10_’) (*N = *41) were used in this study (Table 1). ‘K×K’ population was formed by pooling all the individuals from these three latter F_2_ populations. All these trees are maintained at the collection of the Instituto Valenciano de Investigaciones Agrarias (IVIA) in Valencia (Spain). Additionally, 12 independent F_3_ seed populations (ranging from *N* = 2 to *N* = 77) were obtained after self-pollination of ‘K×K_05_’ and ‘K×K_06_’ trees.

Selfing populations from ‘Katy’ (F_2_ and F_3_) were obtained by putting insect-proof bags over several branches (containing 200–250 flower buds) before anthesis to prevent cross-pollination. Outcrossing populations were obtained by pollinating balloon-stage flowers. Fruits were collected about three months later. F_3_ seed-derived embryos were dissected from the rest of the seed tissue and stored at –20°C.

### Nucleic Acids Extraction

Two leaf discs of each selection were collected and stored at −80°C before DNA isolation. Genomic DNA was extracted following the method of Doyle and Doyle [58]. DNA quantification was performed by NanoDrop ND-1000 spectrophotometer (Thermo Fisher Scientific, Wilmington, DE) and integrity was checked by comparison with lambda DNA (Promega, Madison, WI, USA). Embryo DNA was extracted by incubating for 10 min at 95°C with 20 µl of TPS (100 mM Tris-HCl, pH 9.5; 1 M KCl; 10 mM EDTA) isolation buffer [59]. Total RNA was extracted from mature anthers (contaning mature pollen grains) of balloon-stage flowers using the UltraClean Plant RNA Isolation Kit (MoBio, Carlsbad, CA, USA).

### PCR-amplification, Cloning and Sequencing of *S-RNase* Gene Fragments and the Complete *S*-locus *F-box* Alleles from ‘Katy’

Fragments comprising the *S-RNase* first intron were PCR-amplified with primers SRc-F [44] and Pru-C2R [43] (Table S6) using ‘Katy’ genomic DNA as template. Cycling conditions were as follows: an initial denaturing step of 94°C for 2 min; 30 cycles of 94°C for 30 s, 55°C for 60 s and 72°C for 1 min 30 s; and a final extension of 72°C for 10 min (GeneAmp®PCR System 9700, Perkin-Elmer, Fremont, CA). PCR products were electrophoresed in 1% (w/v) agarose gel, purified using the QIAquick Gel Extraction Kit (Qiagen, Hilden, Germany) and cloned into the pGEM T-Easy vector (Promega, Madison, WI). DNA sequences from four independent clones were determined with an ABI3730 equipment using the Big Dye Terminator v.3.1. cycle sequencing kit (Applied Biosystems, Foster City, CA). Sequences were assembled and edited with the Staden package v1.4 [60] and homology searches were performed with BLASTX [61]. *S-RNase* fragments comprising the second intron were amplified with primers Pru-C2/Pru-C4R [43] (Table S6) using PCR-conditions described by Sonneveld *et al.* [62]. Genomic fragments containing the complete coding sequence of *SFB*_1_ and *SFB*_2_ (as well as their 3′/5′ flanking regions) were PCR-amplified with the haploytpe-specific primer pairs FBf-Hap1/FBr-Hap1 (this work) and FBf-Hap2/FBr-Hap2 [18] respectively (Table S6), using ‘Katy’ genomic DNA as template. PCR conditions and methods for isolating, cloning, and sequencing these fragments were the same used for the *S-RNase* fragments.

### Genomic PCRs for *S*-genotyping

*S*-genotyping of populations and cultivars was performed by PCR-amplification of the *S*-*RNase* first intron with the primer pair SRc-F/SRc-R [44] (Table S6) following the protocol described by Vilanova *et al.* [63].

### Ploidy Level Determination

Ploidy level was determined using the *Partec CyStain UV precise P* reagent kit (Partec PAS, Münster, Germany) for nuclei extraction and DNA staining of nuclear DNA from plant tissues. Approximately 0.5 cm^2^ leaf tissue was chopped using a sharp razor blade in 400 µl extraction buffer and filtered through a *Partec 50 µm CellTrics* disposable filter. Samples were then incubated for 60 seconds in the staining solution and analyzed in the *Partec* flow cytometer Ploidy Analyzer PA (Partec, Münster, Germany) in the blue fluorescence channel.

### Real Time RT-PCR for *SFB*_1_ and *SFB*_2_

cDNA was obtained from total RNA isolated from mature anthers of the cvs. ‘Goldrich’ and ‘Katy’ using the SuperScript III First-Strand Synthesis System for RT-PCR (Invitrogen, Carlsbad, CA, USA). Genomic DNA traces were previously removed from RNA samples by treatment with DNAse I (Invitrogen, Carlsbad, CA, USA). *SFB* allele-specific PCR-primer pairs were designed in this work to amplify *SFB*_1_ and *SFB*_2_ (RT-SFB1-for/RT-SFB1-rev1 and RT-SFB2-for/RT-SFB2-rev2, respectively) (Table S6). Primer allele-specificity was tested by PCR-amplifying both alleles from genomic DNA and comparing fragment sizes with known *S*-genotypes in agarose gels after electrophoresis. The *actin* gene was used as endogenous control and the specific PCR primers Act3 and Act4 designed from the peach genome sequence (Gabino Ríos personal comm.) were used for amplification (Table S6). Specificity of *actin* PCR reaction was tested through size estimation of the amplified product by gel electrophoresis. Real-time PCR reactions were performed using an Applied Biosystems StepOnePlus Real-Time PCR System (Applied Biosystems, Foster City, CA, USA) in a final volume of 20 µl, containing 10 µl of the SYBR Premix Ex Taq (Takara, Foster City, CA, USA), 0.4 µl of ROX reference dye, 0.375 µM of each primer and 2 µl of cDNA template diluted 1∶15 from a total of 20 µl synthesized from 2 µg of total RNA. Cycling conditions were as follows: an initial denaturing step of 95°C for 30 s; 40 cycles of 95°C for 5 s, 60°C for 30 s and 72°C for 1 min. Relative expression of *SFB*_1_ and *SFB*_2_ from ‘Katy’ and ‘Goldrich’ RNA of mature anthers was measured by the standard curve method. Threshold cycle (C_T_) values were automatically determined by StepOne v. 2.0 software (Applied Biosystems, Foster City, CA, USA). PCR reaction specificity was assessed after the amplification by confirming the presence of a single peak in the dissociation curve analysis. Results were the average of three independent biological replicates repeated three times.

### Real-time PCR-based Gene Dosage Assay for *SFB*_1_ and *SFB*_2_

*SFB* allele-specific PCR primers used to determine gene dosage of *SFB*_1_ and *SFB*_2_ from genomic DNA of cvs. ‘Goldrich’ and ‘Katy’ were also RT-SFB1-for/RT-SFB1-rev1 and RT-SFB2-for/RT-SFB2-rev2. *Actin* was used as endogenous control and the specific primers used to amplify this gene were Act3/Act4 (see previous sections). Real-time PCR reactions were performed using the same PCR mixtures (except for 2 µl of gDNA as a template), cycling conditions and thermocycler previously reported for real-time RT-PCR. Relative DNA quantity corresponding to *SFB*_1_ and *SFB*_2_ alleles from ‘Katy’ and ‘Goldrich’ was measured by the standard curve method. C_T_ values and PCR reaction specificity were also determined as for the real-time RT-PCR. Results were the average of two independent biological replicates repeated three times.

### SSR Marker Analysis

A total of 118 SSR markers, spread over the 8 *Prunus* chromosomes, were tested to perform a genome-wide screen for the PPM (Table S7). Those SSRs amplifying in ‘Katy’, ‘Goldrich’ and ‘Canino’ (85) (Table S3) were used to estimate Neís genetic distance between the three cultivars [64] by means of GENETIX v.4.05 software [65]. One hundred and two additional SSRs developed by Zuriaga *et al.* [40] were tested to construct the ‘Katy’ LG3 map (Table S2). SSR amplifications were performed in a GeneAmp® PCR System 9700 thermal cycler (Perkin–Elmer, Freemont, CA, USA) in a final volume of 20 µl, containing 75 mM Tris–HCl, pH 8.8; 20 mM (NH_4_)_2_SO_4_; 1.5 mM MgCl_2_; 0.1 mM of each dNTP; 20 ng of genomic DNA and 1 U of Taq polymerase (Invitrogen, Carlsbad, CA). Each polymerase chain reaction was performed by the procedure of Schuelke [66] using three primers: the specific forward primer of each microsatellite with M13(-21) tail at its 5′ end at 0.4 µM, the sequence-specific reverse primer at 0.8 µM, and the universal fluorescent-labeled M13(-21) primer at 0.4 µM. The following temperature profile was used: 94°C for 2 min, then 35 cycles of 94°C for 45 s, 50–60°C for 1 min, and 72°C for 1 min and 15 s, finishing with 72°C for 5 min. Allele lengths were determined using an ABI Prism 3130 Genetic Analyzer with the aid of ***GeneMapper*** software, version 4.0 (Applied Biosystems).

### *M’*-locus Fine Mapping

Segregation distortion locus (SDL) associated with the PPM was detected using JoinMap 3.0 software [67] by analyzing χ^2^ values of selected SSRs spread over the *Prunus* genome in the ‘K×K_05_’ and ‘K×K_06_’ F_2_ populations. Genetic maps for each linkage group were roughly estimated using these two populations. The logarithm of odds (LOD) grouping threshold was established at ≥ 3.0 for LG2, LG4, LG7 and LG8 but <3.0 for the rest. Comparative mapping with other apricot cvs. was used to support grouping of markers in these latter cases.

Linkage maps of ‘Katy’ chr.3 were constructed using SSR markers segregating in ‘K×K’ and ‘C×K’ populations. Calculations were performed by JoinMap 3.0 software [67] using the Kosambi mapping function [68] to convert recombination units into genetic distances. In the ‘C×K’ population, LG3 was established following the “two-way pseudo test-cross” model of analysis Grattapaglia and Sederoff [69] under a LOD grouping threshold of 5.0 and a recombination frequency parameter below 0.4. According to the single LG3 map obtained for ‘Katy’ from ‘C×K’, LOD score was relaxed to 2.0 for merging, two separated groups (at LOD >5.0) in the ‘K×K’ population to construct LG3.

*M’*-locus genotyping of K×K-F_2_ individuals was indirectly performed by analyzing segregation ratios of heterozygous SSR markers linked to the PPM (according to the SDL analysis) in the F_3_ progenies. A χ^2^ test was performed to check whether the observed ratios fit a 1∶2:1 ratio, corresponding to the *m’m’* genotype, or a 1∶1 ratio, corresponding to the *M’m’* genotype.

## Supporting Information

Table S1 **Identification of segregation distortion SSR loci distributed throughout the ‘Katy’ LG6 using the F_2_ population ‘K×K’.** χ^2^ and *P* values estimated for each SSR, considering the expected segregation ratio 1∶2:1 are indicated.
(DOC)

Table S2 **SSR primers developed from the peach genomic sequence corresponding to the scaffold _3.** Primer position on the scaffold (Mb) and SSR allele sizes amplified in apricot cvs. ‘Goldrich’, ‘Canino’ and ‘Katy’ are indicated.
(DOC)

Table S3 **SSR allele composition for apricot cvs. ‘Goldrich’, ‘Canino’ and ‘Katy’.** Start position on the corresponding scaffold (Number_Mb) and SSR allele sizes (bp) are indicated.
(DOC)

Table S4 **Genetic distances among apricot cvs.** ‘Katy’, ‘Canino’ and ‘Goldrich’ estimated according to Nei [64] (below diagonal) and % of shared SSR alleles (above diagonal).
(DOC)

Table S5 **Gene content of the** ***M’*** **-locus peach syntenic region.** Position and length of the ORFs as well as the first BLASTP match on the TAIR database annotated by IPGI are shown. Overlap length (amino acids), percent id and E-value are indicated for each *Prunus*/*Arabidopsis* gene pair. *Arabidopsis* homologues with detectable expression in mature pollen, hydrated pollen and pollen tubes (+/−) and those with altered transcription during pollen germination (PG) or pollen tube growth (PTG) are also indicated according to the results reported by Wang et al. (2008) using Affymetrix ATH1 Genome Arrays.
(DOC)

Table S6 **Primers used in this study to amplify by PCR different fragments corresponding to** ***S-RNase*** **,** ***SFB*** **and** ***actin*** **genes.**
(DOC)

Table S7 SSR markers tested for the PPM screening on the whole ‘Katy’ genome.
(DOC)

## References

[1] De Nettancourt D (2001) Incompatibility and incongruity in wild and cultivated plants. Springer-Verlag, Berlin. 322 p.

[2] McClure BA, Haring V, Ebert PR, Anderson MA, Simpson RJ, et al (1989) Style self-incompatibility gene products of *Nicotiana alata* are ribonucleases. Nature 342: 955–957. 2594090 10.1038/342955a0

[3] Boskovic R, Tobutt KR (1996) Correlation of stylar ribonuclease zymograms with incompatibility alleles in sweet cherry. Euphytica 90: 245–250.

[4] Xue Y, Carpenter R, Dickinson HG, Coen ES (1996) Origin of allelic diversity in *Antirrhinum S* locus RNases. Plant Cell 8: 805–814. 8672882 10.1105/tpc.8.5.805 PMC161139

[5] Lai Z, Ma W, Han B, Liang L, Zhang Y, et al (2002) An F-box gene linked to the self-incompatibility (*S*) locus of *Antirrhinum* is expressed specifically in pollen and tapetum. Plant Mol Biol 50: 29–42. 12139007 10.1023/a:1016050018779

[6] Sijacic P, Wang X, Skirpan AL, Wang Y, Dowd PE, et al (2004) Identification of the pollen determinant of S-RNase-mediated self-incompatibility. Nature 429: 302–305. 15152253 10.1038/nature02523

[7] Ushijima K, Sassa H, Dandekar AM, Gradziel TM, Tao R, et al (2003) Structural and transcriptional analysis of the self-incompatibility locus of almond: Identification of a pollen-expressed F-box gene with haplotype-specific polymorphism. Plant Cell 15: 771–781. 12615948 10.1105/tpc.009290 PMC150029

[8] Hua Z, Kao TH (2006) Identification and characterization of components of a putative *Petunia S*-locus F-box-containing E3 ligase complex involved in S-RNase based self-incompatibility. Plant Cell 18: 2531–2553. 17028207 10.1105/tpc.106.041061 PMC1626602

[9] Huang J, Zhao L, Yang Q, Xue Y (2006) AhSSK1, a novel SKP1-like protein that interacts with the S-locus F-box protein SLF. Plant J 46: 780–793. 16709194 10.1111/j.1365-313X.2006.02735.x

[10] Goldraij A, Kondo K, Lee CB, Hancock CN, Sivaguru M, et al (2006) Compartmentalization of S-RNase and HT-B degradation in self-incompatible *Nicotiana* . Nature 439: 805–810. 16482149 10.1038/nature04491

[11] Chen G, Zhang B, Zhao Z, Sui Z, Zhang H, et al (2010) ‘A life or death decision’ for pollen tubes in S-RNase-based self-incompatibility. J Exp Bot 61: 2027–2037. 20042540 10.1093/jxb/erp381

[12] Yamane H, Ikeda K, Hauck NR, Iezzoni AF, Tao R (2003) Self-incompatibility (*S*) locus region of the mutated *S* ^6^-haplotype of sour cherry (*Prunus cerasus*) contains a functional pollen *S* allele and a non-functional pistil *S* allele. J Exp Bot 54: 2431–2437. 14512382 10.1093/jxb/erg271

[13] Watari A, Hanada T, Yamane H, Esumi T, Tao R, et al (2007) A low transcriptional level of *Se*-RNase in the *Se*-haplotype confers self-compatibility in Japanese plum. J Am Soc Hort Sci 132: 396–406.

[14] Tao R, Watari A, Hanada T, Habu T, Yaegaki H, et al (2007) Self-compatible peach (*Prunus persica*) has mutant versions of the S haplotypes found in self-incompatible *Prunus* species. Plant Mol Biol 63: 109–123. 17006593 10.1007/s11103-006-9076-0

[15] Ushijima K, Yamane H, Watari A, Kakehi E, Ikeda K, et al (2004) The *S* haplotype-specific F-box protein gene, *SFB*, is defective in self-compatible haplotypes of *Prunus avium* and *P. mume* . Plant J 39: 573–586. 15272875 10.1111/j.1365-313X.2004.02154.x

[16] Sonneveld T, Tobbutt KR, Vaughan SP, Robbins TP (2005) Loss of pollen-*S* function in two self-compatible selections of *Prunus avium* is associated with deletion/mutation of an *S* haplotype-specific F-box gene. Plant Cell 17: 37–51. 15598801 10.1105/tpc.104.026963 PMC544488

[17] Marchese A, Boskovic R, Caruso T, Raimondo A, Cutuli M, et al (2007) A new self-incompatibility haplotype in sweet cherry ‘Kronio’, S_5_′ attributable to a pollen-part mutation in the SFB gene. J Exp Bot 58: 4347–4356. 18182436 10.1093/jxb/erm322

[18] Vilanova S, Badenes ML, Burgos L, Martínez-Calvo J, Llácer G, et al (2006) Self-compatibility of two apricot selections is associated with two pollen-part mutations of different nature. Plant Physiol 42: 629–641. 10.1104/pp.106.083865 PMC1586032 16920873

[19] Hauck NR, Yamane H, Tao R, Iezzoni AF (2006) Accumulation of non-functional S-haplotypes results in the breakdown of gametophytic self-incompatibility in tetraploid Prunus. Genetics 172: 1191–1198. 16219786 10.1534/genetics.105.049395 PMC1456217

[20] Yamane H, Tao R (2009) Molecular basis of self-(in)compatibility and current status of *S*-genotyping in Rosaceous fruit trees. J Jpn Soc Hort Sci 78: 137–157.

[21] Golz JF, Su V, Clarke AE, Newbigin E (1999) A molecular description of mutations affecting the pollen counterpart of the *Nicotiana alata S* locus. Genetics 152: 1123–1135. 10388830 10.1093/genetics/152.3.1123 PMC1460670

[22] Kubo K, Entani T, Tanaka A, Wang N, Fields AM, et al (2010) Collaborative non-self recognition system in S-RNase-based self-incompatibility. Science 330: 796–799. 21051632 10.1126/science.1195243

[23] Kakui H, Kato M, Ushijima K, Kitaguchi M, Kato S, et al (2011) Sequence divergence and loss-of-function phenotypes of S locus F-box brothers genes are consistent with non-self recognition by multiple pollen determinants in self-incompatibility of Japanese pear (*Pyrus pyrifolia*). Plant J 68: 1028–1038. 21851432 10.1111/j.1365-313X.2011.04752.x

[24] Tao R, Iezzoni AF (2010) The S-RNase-based gametophytic self-incompatibility system in *Prunus* exhibits distinct genetic molecular features. Sci Hort 124: 423–433.

[25] McClure B, Cruz-García F, Romero C (2011) Compatibility and incompatibility in S-RNase-based systems. Ann Bot 108: 647–658. 21803740 10.1093/aob/mcr179 PMC3170157

[26] Wünsch A, Hormaza JI (2004) Genetic and molecular analysis in Cristobalina sweet cherry, a spontaneous self-compatible mutant. Sex Plant Reprod 17: 203–210.

[27] Fernández A, Hanada T, Alonso JM, Yamane H, Tao R, et al (2009) A modifier locus affecting the expression of the S-RNase gene could be the cause of breakdown of self-incompatibility in almond. Sex Plant Reprod 22: 179–186. 20033438 10.1007/s00497-009-0102-7

[28] Boskovic R, Sargent DJ, Tobutt KR (2010) Genetic evidence that two independent *S*-loci control RNase-based self-incompatibility in diploid strawberry. J Exp Bot 61: 755–763. 20008462 10.1093/jxb/erp340 PMC2814107

[29] Ai Y, Kron E, Kao TH (1991) S-alleles are retained and expressed in a self-compatible cultivar of *Petunia hybrida* . Mol Gen Genet 230: 353–358. 1766433 10.1007/BF00280291

[30] McClure BA, Cruz-García F, Beecher B, Sulaman W (2000) Factors affecting inter- and intra-specific pollen rejection in *Nicotiana* . Ann Bot 85: 113–123.

[31] Thompson RD, Uhrig H, Hermsen JGT, Salamini F, Kaufmann H (1991) Investigation of a self-compatible mutation in *Solanum tuberosum* clones inhibiting S-allele activity in pollen differentially. Mol Gen Genom 226: 283–288. 10.1007/BF00273614 1851951

[32] Tsukamoto T, Ando T, Takahashi K, Omori T, Watanabe H, et al (2003) Breakdown of self-incompatibility in a natural population of Petunia axillaris caused by loss of pollen function. Plant Physiol 131: 1903–1912. 12692349 10.1104/pp.102.018069 PMC166946

[33] Hosaka K, Hanneman RE (1998) Genetics of self-compatibility in a selfcompatible wild diploid potato species *Solanum chacoense*.1. Detection of an *S*-locus inhibitor (*Sli*) gene. Euphytica 99: 191–197.

[34] Hosaka K, Hanneman RE (1998) Genetics of self-compatibility in a selfcompatible wild diploid potato species *Solanum chacoense*. 2. Localization of an *S*-locus inhibitor (*Sli*) gene on the potato genome using DNA markers. Euphytica 103: 265–271.

[35] McClure B, Mou B, Canevascini S, Bernatzky R (1999) A small asparagine-rich protein required for *S*-allele-specific pollen rejection in *Nicotiana* . Proc Natl Acad Sci USA 96: 13548–13553. 10557358 10.1073/pnas.96.23.13548 PMC23985

[36] Hancock CN, Kent L, McClure BA (2005) The 120 kDa glycoprotein is required for *S*-specific pollen rejection in *Nicotiana* . Plant J 43: 716–723. 16115068 10.1111/j.1365-313X.2005.02490.x

[37] Busot GY, McClure B, Ibarra-Sánchez CP, Jiménez-Durán K, Vázquez-Santana S, et al (2008) Pollination in *Nicotiana alata* stimulates synthesis and transfer to the stigmatic surface of NaStEP, a vacuolar Kunitz proteinase inhibitor homologue. J Exp Bot 59: 3187–3201. 18689443 10.1093/jxb/ern175 PMC2504342

[38] Zhao L, Huang J, Zhao Z, Li Q, Sims TL, et al (2010) The Skp-like protein SSK is required for cross-pollen compatibility in S-RNase-based self-incompatibility. Plant J 62: 52–63. 20070569 10.1111/j.1365-313X.2010.04123.x

[39] Matsumoto D, Yamane H, Abe K, Tao R (2012) Identification of a Skp1-like protein interacting with SFB, the pollen *S* determinant of the gametophytic self-incompatibility in *Prunus.* . Plant Physiol 159: 1252–1262. 22548785 10.1104/pp.112.197343 PMC3387707

[40] Zuriaga E, Molina L, Badenes ML, Romero C (2012) Physical mapping of a pollen modifier locus controlling self-incompatibility in apricot and synteny analysis within the Rosaceae. Plant Mol Biol 79: 229–242. 22481163 10.1007/s11103-012-9908-z

[41] Russell D (1998) The stonefruit cultivar system (a database of worldwide stonefruit cultivars and rootstocks). Department of Primary Industries, Queensland, Australia.

[42] Burgos L, Pérez-Tornero O, Ballester J, Olmos E (1998) Detection and inheritance of stylar ribonucleases associated with incompatibility alleles in apricot. Sex Plant Reprod 11: 153–158.

[43] Tao R, Yamane H, Sugiura A (1999) Molecular typing of S-alleles through identification, characterization and cDNA cloning for S-RNAses in sweet cherry. J Am Soc Hort Sci 124: 224–233.

[44] Romero C, Vilanova S, Burgos L, Martínez-Calvo J, Vicente M, et al (2004) Analysis of the *S*-locus structure in *Prunus armeniaca* L. Identification of *S*-haplotype specific *S-RNase* and *F-box* genes. Plant Mol Biol 56: 145–157. 15604734 10.1007/s11103-004-2651-3

[45] Golz JF, Oh HY, Su V, Kusaba M, Newbigin E (2001) Genetic analysis of *Nicotiana* pollen-part mutants is consistent with the presence of an S-ribonuclease inhibitor at the *S* locus. Proc Natl Acad Sci USA 98: 15372–15376. 11752474 10.1073/pnas.261571598 PMC65036

[46] Zhebentyayeva TN, Swire-Clark G, Georgi LL, Garay L, Jung S, et al (2008) A framework physical map for peach, a model Rosaceae species. Tree Genet Genomes 4: 745–756.

[47] Jung S, Jesudurai C, Staton M, Du Z, Ficklin S, et al (2004) GDR (Genome Database for Rosaceae): integrated web resources for Rosaceae genomics and genetics research. BMC Bioinformatics 5: 130. 15357877 10.1186/1471-2105-5-130 PMC517928

[48] Wang Y, Zhang WZ, Song LF, Zou JJ, Su Z, et al (2008) Transcriptome analyses show changes in gene expression to accompany pollen germination and tube growth in *Arabidopsis* . Plant Physiol 148: 1201–1211. 18775970 10.1104/pp.108.126375 PMC2577266

[49] Feng J, Chen X, Wu Y, Liu W, Liang Q, et al (2006) Detection and transcript expression of S-RNase gene associated with self-incompatibility in apricot (*Prunus armeniaca* L.). Mol Biol Rep 33: 215–221. 16850191 10.1007/s11033-006-0011-x

[50] Wu J, Gu C, Du YH, Wu HQ, Liu WS, et al (2010) Self-compatibility of ‘Katy’ apricot (*Prunus armeniaca* L.) is associated with pollen part mutations. Sex Plant Reprod 24: 23–35. 20658154 10.1007/s00497-010-0148-6

[51] Egea J, Burgos L (1996) Detecting cross-incompatibility of three North American apricot cultivars and establishing the first incompatibility group in apricot. J Am Soc Hort Sci 12: 1002–1005.

[52] Alburquerque N, Egea J, Pérez-Tornero O, Burgos L (2002) Genotyping apricot cultivars for self-(in)compatibility by means of RNases associated with S alleles. Plant Breed 121: 343–347.

[53] Wünsch A, Tao R, Hormaza JI (2010) Self-compatibility in ‘Cristobalina’ sweet cherry is not associated with duplications or modified transcription levels of *S*-locus genes. Plant Cell Rep 29: 715–721. 20411390 10.1007/s00299-010-0857-1

[54] Zhu C, Zhang YM (2007) An EM algorithm for mapping distortion segregation loci. BMC Genetics 8: 82. 18047652 10.1186/1471-2156-8-82 PMC2257974

[55] Cachi AM, Wünsch A (2011) Characterization and mapping of non-*S* gametophytic self-compatibility in sweet cherry (*Prunus avium* L.). J Exp Bot 62: 1847–1856. 21127024 10.1093/jxb/erq374

[56] Zheng XH, Lu F, Wang ZY, Zhong F, Hoover J, et al (2005) Using shared genomic synteny and shared protein functions to enhance the identification of orthologous gene pairs. Bioinformatics 21: 703–710. 15458983 10.1093/bioinformatics/bti045

[57] Movahedi S, Van de Peer Y, Vandepoele K (2011) Comparative network analysis reveals that tissue specificity and gene function are important factors influencing the mode of expression evolution in *Arabidopsis* and rice. Plant Physiol 156: 1316–1330. 21571672 10.1104/pp.111.177865 PMC3135928

[58] Doyle JJ, Doyle JL (1987) A rapid isolation procedure for small quantities of fresh leaf tissue. Phyto Bull 19: 11–15.

[59] Thomson D, Henry R (1995) Single-step protocol for preparation of plant tissue for analysis by PCR. Biotechniques 19: 394–400. 7495552

[60] Bonfield J (2004) Staden package, version 1.4. Available: http://staden.sourceforge.net Accessed 2011 July 27 July.

[61] Altschul SF, Gish W, Miller W, Myers EW, Lipman DJ (1990) Basic local alignment search tool. J Mol Biol 215: 403–410. 2231712 10.1016/S0022-2836(05)80360-2

[62] Sonneveld T, Tobutt KR, Robbins TP (2003) Allele-specific PCR detection of sweet cherry self-incompatibility (S) alleles S1 to S16 using consensus and allele-specific primers. Theor Appl Genet 107: 1059–1070. 14523529 10.1007/s00122-003-1274-4

[63] Vilanova S, Romero C, Burgos L, Llácer G, Badenes ML (2005) Identification of self-(in)compatiblity alleles in apricot (*Prunus armeniaca* L.) by PCR and sequence analysis. J Am Soc Hort Sci 130: 893–898.

[64] Nei M (1972) Genetic distance between populations. Am Nat 106: 283–292.

[65] Belkhir K, Borsa P, Chikhi L, Raufaste N, Bonhomme F (2004) GENETIX 4.05, logiciel sous Windows TM pour la génétique des populations. Laboratoire Génome, Populations, Interactions, CNRS UMR 5171, Université de Montpellier II, Montpellier (France).

[66] Schuelke M (2000) An economic method for the fluorescent labelling of PCR fragments. Nat Biotechnol 18: 233–234. 10657137 10.1038/72708

[67] Van Ooijen JW, Voorrips RE (2001) JoinMap®3.0, Software for the calculation of genetic linkage maps. Plant Research International, Wageningen, The Netherlands.

[68] Kosambi DD (1944) The estimation of map distance from recombination values. Ann Eugen 12: 172–175.

[69] Grattapaglia D, Sederoff RR (1994) Genetic linkage maps of *Eucalyptus grandis* and *E. urophylla* using a pseudotest-cross strategy and RAPD markers. Genetics 137: 1121–1137. 7982566 10.1093/genetics/137.4.1121 PMC1206059

