# Supplementary material for: An S-Locus Independent Pollen Factor Confers Self-Compatibility in ‘Katy’ Apricot
Source: PLoS One. 2013 Jan 14;8(1):e53947. doi: 10.1371/journal.pone.0053947 (PMC3544744; doi:10.1371/journal.pone.0053947)
Supplement: Table S1 — Identification of segregation distortion SSR loci distributed throughout the ‘Katy’ LG6 using the F2 population ‘K×K’. χ2 and P values estimated for each SSR, considering the expected segregation ratio 1∶2:1 are indicated. (DOC) [file pone.0053947.s001.doc]

**Table S1 Identification of segregation distortion SSR loci distributed throughout the ‘Katy’ LG6 using the F_2_ population ‘K×K’.** χ^2^ and *P* values estimated for each SSR, considering the expected segregation ratio 1:2:1 are indicated.

| **LG** | **Locus** | **Peach Mb^a^** | **Seg. Type^b^** | **A** | **H** | **B** | Total | **χ^2^ (*P*-value)^c^** |
| --- | --- | --- | --- | --- | --- | --- | --- | --- |
| 6 | PGS6_01 | 00,12 | <abxab> | 28 | 45 | 12 | 85 | 6,32 (0,04)^d^ |
| 6 | PGS6_04 | 04,95 | <abxab> | 29 | 45 | 11 | 85 | 7,92 (0,02)^d^ |
| 6 | UDAp420 | 08,14 | <abxab> | 34 | 41 | 12 | 87 | 11,41 (0,03)^d^ |
| 6 | PGS6_07 | 09,33 | <abxab> | 32 | 47 | 7 | 86 | 15,28 (0,0004)^d^ |
| 6 | UDAp489 | 16,82 | <abxab> | 34 | 41 | 11 | 86 | 12,49 (0,002)^d^ |
| 6 | Ma027a | 20,90 | <abxab> | 33 | 43 | 9 | 85 | 13,57 (0,001)^d^ |
| 6 | BBPCT025 | 21,13 | <abxab> | 35 | 42 | 10 | 87 | 14,47 (0,0007)^d^ |
| 6 | UDP98-412 | 24,75 | <abxab> | 28 | 43 | 12 | 83 | 6,28 (0,04)^d^ |
| 6 | Locus-S | 26,45 | <abxab> | 31 | 40 | 16 | 87 | 5,74 (0,06) |
| 6 | ssrPaCITA12 | 27,84 | <abxab> | 31 | 38 | 16 | 85 | 6,24 (0,04)^d^ |

^a^ Marker position (Mb) within the peach genome scaffold_6 which size estimated by IPGI was 28.90 Mb

^b^ Segregation type as per JoinMap 3.0

^c^ Chi-square test was performed for the expected ratio 1:2:1 (<abxab>)

^d^ Observed ratios differ significantly from expected at *P* < 0.05 for 2 degrees of freedom
