# Supplementary material for: An S-Locus Independent Pollen Factor Confers Self-Compatibility in ‘Katy’ Apricot
Source: PLoS One. 2013 Jan 14;8(1):e53947. doi: 10.1371/journal.pone.0053947 (PMC3544744; doi:10.1371/journal.pone.0053947)
Supplement: Table S2 — SSR primers developed from the peach genomic sequence corresponding to the scaffold _3. Primer position on the scaffold (Mb) and SSR allele sizes amplified in apricot cvs. ‘Goldrich’, ‘Canino’ and ‘Katy’ are indicated. (DOC) [file pone.0053947.s002.doc]

**Table S2 SSR primers developed from the peach genomic sequence corresponding to the scaffold _3.** Primer position on the scaffold (Mb) and SSR allele sizes amplified in apricot cvs. ‘Goldrich’, ‘Canino’ and ‘Katy’ are indicated.

| Name | Start on scaffold_3 | Goldrich  alleles | Canino  alleles | Katy  alleles | Name | Start on scaffold_3 | Goldrich  alleles | Canino  alleles | Katy  alleles |
| --- | --- | --- | --- | --- | --- | --- | --- | --- | --- |
| PGS3.01 | 16,21 | 176 | 168/176 | N.A. | PGS3.52 | 17,96 | 194/208 | 194 | 194/204 |
| PGS3.02 | 16,30 | 163/174 | 166/174 | 163/174 | PGS3.53 | 18,07 | 199/203 | 199 | 197/199 |
| PGS3.03 | 16,41 | 162/178 | 156/162 | 162/178 | PGS3.54 | 18,12 | 280/282 | 282 | 262/282 |
| PGS3.04 | 16,52 | N.A.^a^ | N.A. | N.A. | PGS3.55 | 18,19 | 449 | 449 | N.A. |
| PGS3.05 | 16,64 | 171 | 171 | N.A. | PGS3.56 | 18,22 | 251/258 | 258 | 251/267 |
| PGS3.06 | 16,70 | 152/160 | 152 | ML | PGS3.57 | 18,23 | N.A. | N.A. | N.A. |
| PGS3.07 | 16,84 | 141 | 141 | 141 | PGS3.58 | 18,24 | 95/99 | 95 | N.A. |
| PGS3.08 | 16,91 | 157 | 157 | 157 | PGS3.59 | 18,50 | 211/215 | 215 | 215/217 |
| PGS3.09 | 17,01 | 204 | 204 | 204 | PGS3.60 | 18,51 | 293/317 | 317 | N.A. |
| PGS3.10 | 17,16 | 130 | 129/130 | 129 | PGS3.61 | 18,58 | 173 | 173 | 173 |
| PGS3.11 | 17,22 | N.A. | 156 | N.A. | PGS3.62 | 18,61 | 336/350 | 348/350 | N.A. |
| PGS3.12 | 17,38 | 148 | 148/162 | 148/156 | PGS3.63 | 18,65 | 218/220 | 220 | 212/220 |
| PGS3.13 | 17,54 | 230 | 202/230 | 215/230 | PGS3.64 | 18,70 | ML | ML | ML |
| PGS3.14 | 17,63 | N.A. | N.A. | N.A. | PGS3.65 | 18,31 | ML | ML | ML |
| PGS3.15 | 17,71 | 266/267 | 267/274 | 266/267 | PGS3.66 | 18,34 | 322/328 | 328 | 322/328 |
| PGS3.16 | 17,80 | N.A. | N.A. | N.A. | PGS3.67 | 18,35 | 210/216 | 210 | 201/210 |
| PGS3.17 | 17,98 | N.A. | N.A. | N.A. | PGS3.68 | 18,37 | 311/338 | 338 | 338 |
| PGS3.18 | 18,06 | 147 | 147 | 147 | PGS3.69 | 18,38 | 202/227 | 202 | N.A. |
| PGS3.19 | 18,12 | 147 | 199 | N.A. | PGS3.70 | 18,40 | 200/201 | 200 | 198/200 |
| PGS3.20 | 18,25 | 148 | N.A. | 150 | PGS3.71 | 18,40 | 255/259 | 259/261 | 245/259 |
| PGS3.21 | 18,40 | 229/244 | 244 | 217 | PGS3.72 | 18,41 | ML | ML | ML |
| PGS3.22 | 18,49 | 306/310 | 306 | 306/312 | PGS3.73 | 18,43 | 368 | 368 | 368 |
| PGS3.23 | 18,61 | 179/188 | 188 | 188/190 | PGS3.74 | 18,46 | 262/265 | 265 | 265 |
| PGS3.24 | 18,77 | ML^b^ | ML | ML | PGS3.75 | 18,48 | N.A. | N.A. | 351/352 |
| PGS3.25 | 18,87 | 180/192 | 186/192 | 186 | PGS3.76 | 18,49 | 192 | 192 | 192/198 |
| PGS3.26 | 18,94 | 155/166 | 160/166 | ML | PGS3.77 | 18,50 | 273/276 | 273 | 273/276 |
| PGS3.27 | 19,03 | N.A. | N.A. | N.A. | PGS3.78 | 18,54 | 161/164 | 164 | 164/166 |
| PGS3.28 | 19,14 | 141 | 141 | 141/143 | PGS3.79 | 18,57 | 460 | 460 | N.A. |
| PGS3.29 | 19,25 | 159 | 159 | 159 | PGS3.80 | 18,59 | 474 | 474 | N.A. |
| PGS3.30 | 19,34 | 247 | 242 | 242 | PGS3.81 | 18,61 | 227 | 227 | 227 |
| PGS3.31 | 19,45 | ML | ML | ML | PGS3.82 | 18,61 | 148 | 148 | 148 |
| PGS3.32 | 19,60 | 256 | 256 | 256/270 | PGS3.83 | 18,61 | ML | ML | N.A. |
| PGS3.33 | 19,66 | 133/139 | 133/139 | 129/133 | PGS3.84 | 18,61 | N.A. | N.A. | N.A. |
| PGS3.34 | 17,75 | 185/191 | 185/193 | N.A. | PGS3.85 | 18,62 | ML | ML | 197/199 |
| PGS3.35 | 17,85 | N.A. | N.A. | N.A. | PGS3.86 | 18,63 | 170/179 | 170 | 170/179 |
| PGS3.36 | 17,95 | 148/162 | 325/327 | N.A. | PGS3.87 | 18,63 | 335/340 | 340 | N.A. |
| PGS3.37 | 18,00 | 189/202 | 189 | 189/202 | PGS3.88 | 18,65 | 278 | N.A. | 284 |
| PGS3.38 | 18,05 | 246/264 | 264 | 264 | PGS3.89 | 18,66 | 404/405 | 405 | 405 |
| PGS3.39 | 18,07 | 165 | 165 | 165 | PGS3.90 | 18,69 | ML | ML | 185 |
| PGS3.40 | 18,14 | 128 | 177 | 177 | PGS3.91 | 18,70 | 285/null | N.A. | N.A. |
| PGS3.41 | 18,18 | N.A. | N.A. | N.A. | PGS3.92 | 18,70 | ML | ML | ML |
| PGS3.42 | 18,22 | 206/224 | 206 | 206/209 | PGS3.93 | 18,73 | 206/207 | 206 | 206 |
| PGS3.43 | 18,24 | N.A. | N.A. | N.A. | PGS3.94 | 18,74 | N.A. | N.A. | N.A. |
| PGS3.44 | 18,29 | 295/307 | 307/309 | 264/309 | PGS3.95 | 18,75 | 344 | 344 | 344 |
| PGS3.45 | 18,33 | 237 | N.A. | 237 | PGS3.96 | 18,76 | 434/441 | 441/442 | N.A. |
| PGS3.46 | 18,47 | 163/173 | 163 | 163/181 | PGS3.97 | 18,80 | N.A. | N.A. | 180/237 |
| PGS3.47 | 18,52 | 242/246 | 242 | N.A. | PGS3.98 | 18,81 | N.A. | N.A. | N.A. |
| PGS3.48 | 18,60 | 256/264 | 264 | 256/264 | PGS3.99 | 18,81 | N.A. | N.A. | N.A. |
| PGS3.49 | 18,63 | N.A. | N.A. | N.A. | PGS3.100 | 18,84 | 239 | 239 | 239 |
| PGS3.50 | 18,83 | N.A. | N.A. | N.A. | PGS3.101 | 18,84 | N.A. | N.A. | 222 |
| PGS3.51 | 17,83 | ML | ML | 149/192 | PGS3.102 | 18,85 | 251 | 251 | 251 |

^a^ N.A. Not amplified

^b^ ML. Multi-loci pattern
