# Supplementary material for: An S-Locus Independent Pollen Factor Confers Self-Compatibility in ‘Katy’ Apricot
Source: PLoS One. 2013 Jan 14;8(1):e53947. doi: 10.1371/journal.pone.0053947 (PMC3544744; doi:10.1371/journal.pone.0053947)
Supplement: Table S3 — SSR allele composition for apricot cvs. ‘Goldrich’, ‘Canino’ and ‘Katy’. Start position on the corresponding scaffold (Number_Mb) and SSR allele sizes (bp) are indicated. (DOC) [file pone.0053947.s003.doc]

**Table S3 SSR allele composition for apricot cvs. ‘Goldrich’, ‘Canino’ and ‘Katy’.** Start position on the corresponding scaffold (Number_Mb) and SSR allele sizes (bp) are indicated.

| Name | Start on scaffold | Goldrich  alleles | Canino  alleles | Katy  alleles | Name | Start on scaffold | Goldrich  alleles | Canino  alleles | Katy  alleles |
| --- | --- | --- | --- | --- | --- | --- | --- | --- | --- |
| Gol051 | 1_04,69 | 177/181 | 181/183 | 181/183 | PGS4.05 | 4_12,03 | 171/171 | 171/171 | 171/171 |
| EPPUC0027 | 1_09,51 | 174/176 | 170/176 | 170/174 | PGS4.07 | 4_18,14 | 179/179 | 176/176 | 179/179 |
| pchcms4 | 1_09,51 | 246/248 | 242/248 | 242/246 | UDAp404 | 4_-- | 165/165 | 188/188 | 165/177 |
| UDAp414 | 1_26,52 | 179/183 | 167/187 | 179/179 | PGS5.02 | 5_00,48 | 178/183 | 183/183 | 178/181 |
| EPPCU1589 | 1_31,81 | 160/176 | 176/182 | 182/182 | PGS5.03 | 5_05,06 | 138/138 | 136/138 | 138/138 |
| CPPCT045 | 1_32.02 | 125/134 | 125/128 | 128/134 | SsrPaCITA21 | 5_10,78 | 241/241 | 239/241 | 241/241 |
| SsrPaCITA7 | 1_32,02 | 221/227 | 209/227 | 227/227 | CPSCT006 | 5_11,53 | 136/138 | 136/138 | 136/140 |
| Gol004 | 1_45,40 | 221/231 | 208/221 | 231/231 | BPPCT037 | 5_12,31 | 132/137 | 153/163 | 132/132 |
| SsrPaCITA16 | 2_03,76 | 130/130 | 130/152 | 130/130 | pchgms4 | 5_12,67 | 168/168 | 168/195 | 168/168 |
| PGS2.03 | 2_04,36 | 177/177 | 177/177 | 177/177 | UDAp452 | 5_13,76 | 190/197 | 190/197 | 189/201 |
| SsrPaCITA19 | 2_13,01 | 156/167 | 132/167 | 156/167 | PGS5.08 | 5_15,30 | 219/219 | 219/219 | 219/219 |
| CPSCT038 | 2_14,47 | 207/207 | 216/226 | 207/207 | PGS6.01 | 6_00,12 | 284/289 | 289/289 | 284/289 |
| BPPCT001 | 2_16,13 | 115/116 | 115/116 | 115/115 | PGS6.02 | 6_01,14 | 265/269 | 263/263 | 255/263 |
| CPSCT044 | 2_17,22 | 209/211 | 199/211 | 209/209 | PGS6.03 | 6_01,23 | 221/225 | 227/227 | 227/233 |
| UDP98-411 | 2_20,17 | 177/184 | 181/192 | 181/184 | PGS6.04 | 6_04,95 | 149/158 | 149/155 | 149/158 |
| CPSCT021 | 2_23,74 | 148/156 | 148/152 | 152/156 | UDAp420 | 6_08,14 | 187/196 | 187/196 | 185/187 |
| CPSCT031 | 2_25,15 | 203/205 | 205/205 | 203/205 | PGS6.07 | 6_09,33 | 183/185 | 185/201 | 186/196 |
| CPSCT023 | 2_25,34 | 210/235 | 216/216 | 210/216 | PGS6.08 | 6_09,35 | 236/238 | 238/238 | 228/238 |
| CPSCT034 | 2_26,35 | 216/231 | 212/231 | 216/216 | BPPCT008 | 6_10,28 | 86/107 | 86/105 | 86/113 |
| MA066a | 3_02,40 | 131/131 | 125/125 | 131/133 | UDAp489 | 6_16,82 | 164/181 | 181/181 | 148/181 |
| SsrPaCITA23 | 3_02,70 | 157/161 | 155/165 | 157/165 | Ma027a | 6_20,90 | 148/180 | 152/170 | 154/180 |
| BPPCT007 | 3_02,74 | 185/185 | 165/171 | 165/165 | BPPCT025 | 6_21,13 | 150/160 | 148/160 | 150/160 |
| UDAp446 | 3_04.50 | 166/179 | 166/168 | 166/179 | UDP98-412 | 6_24,75 | 104/110 | 82/110 | 82/104 |
| UDAp468 | 3_04,85 | 170/182 | 158/170 | 170/182 | Locus-*S* | 6_26,45 | S1/S2 | S2/SC | S1/S2 |
| BPPCT039 | 3_05,80 | 182/187 | 165/187 | 182/187 | SsrPaCITA12 | 6_27,84 | 162/168 | 165/168 | 162/168 |
| EPPCU2256 | 3_06,14 | 162/254 | 162/172 | 162/254 | CPSCT004 | 7_6,68 | 143/143 | 143/145 | 143/143 |
| EPDCU3083 | 3_06,46 | 149/149 | 149/151 | 149/149 | CPPCT022 | 7_10,23 | 257/289 | 269/289 | 252/257 |
| UDA002 | 3_10,85 | 175/186 | 147/147 | 175/175 | UDP98-405 | 7_10,94 | 120/120 | 120/124 | 120/120 |
| SsrPaCITA10 | 3_14,16 | 191/193 | 191/191 | 193/193 | CPSCT026 | 7_10,98 | 218/218 | 206/209 | 211/218 |
| UDAp499 | 3_14,71 | 120/239 | 120/266 | 120/239 | PGS7.05 | 7_13,08 | 217/221 | 205/215 | 217/217 |
| SsrPaCITA4 | 3_14,81 | 152/158 | 166/166 | 152/152 | CPPCT033 | 7_16,70 | 142/142 | 142/142 | 138/142 |
| EPPCU9343 | 3_16,70 | 194/202 | 194/194 | 194/202 | CPSCT042 | 7_17,08 | 187/192 | 181/187 | 181/192 |
| EPPCU7190 | 3_19,78 | 212/214 | 212/214 | 214/226 | CPSCT018 | 8_00,12 | 160/160 | 165/166 | 160/160 |
| AMPA119 | 3_20,00 | 114/118 | 114/118 | 118/128 | UDAp423 | 8_00,18 | 151/184 | 151/151 | 151/184 |
| UCDCH19 | 3_20,03 | 143/143 | 135/143 | 133/135 | PGS8.02 | 8_03,92 | 326/343 | 330/330 | 326/343 |
| CPDCT027 | 3_21,67 | 169/null | 166/null | 166/167 | PGS8.05 | 8_07,39 | 251/251 | 243/251 | 245/253 |
| EPPCU0532 | 3_22,00 | 182/182 | 182/186 | 182/184 | UDAp401 | 8_10,50 | 220/222 | 222/222 | 218/222 |
| PGS4.01 | 4_03,46 | 348/348 | 356/356 | 348/356 | UDAp470 | 8_12,61 | 110/120 | 120/120 | 114/120 |
| PGS4.02 | 4_03,49 | 169/175 | 169/169 | 169/175 | CPPCT006 | 8_13,66 | 200/200 | 200/200 | 200/204 |
| CPDCT045 | 4_06,21 | 125/134 | 125/128 | 128/134 | M6a | 8_15,03 | 222/222 | 196/222 | 204/222 |
| BPPCT040 | 4_06,46 | 151/158 | 151/160 | 158/160 | UDP98-409 | 8_17,78 | 148/152 | 152/168 | 148/148 |
| UDP96-003 | 4_08,76 | 112/126 | 112/112 | 112/126 | Ma035a | 8_21,83 | 170/180 | 180/182 | 180/180 |
| PGS4.04 | 4_11,99 | 278/278 | 265/265 | 288/288 |  |  |  |  |  |
