# Supplementary material for: An S-Locus Independent Pollen Factor Confers Self-Compatibility in ‘Katy’ Apricot
Source: PLoS One. 2013 Jan 14;8(1):e53947. doi: 10.1371/journal.pone.0053947 (PMC3544744; doi:10.1371/journal.pone.0053947)
Supplement: Table S4 — Genetic distances among apricot cvs. ‘Katy’, ‘Canino’ and ‘Goldrich’ estimated according to Nei [64] (below diagonal) and % of shared SSR alleles (above diagonal). (DOC) [file pone.0053947.s004.doc]

**Table S4 Genetic distances among apricot cvs. ‘Katy’, ‘Canino’ and ‘Goldrich’ estimated according to Nei [64] (below diagonal) and % of shared SSR alleles (above diagonal).**

|  | Katy | Canino | Goldrich |
| --- | --- | --- | --- |
| Katy | --- | 38,8 | 61,2 |
| Canino | 0,83 | --- | 44,7 |
| Goldrich | 0,39 | 0,73 | --- |
