# Supplementary material for: An S-Locus Independent Pollen Factor Confers Self-Compatibility in ‘Katy’ Apricot
Source: PLoS One. 2013 Jan 14;8(1):e53947. doi: 10.1371/journal.pone.0053947 (PMC3544744; doi:10.1371/journal.pone.0053947)
Supplement: Table S5 — Gene content of the M’ -locus peach syntenic region. Position and length of the ORFs as well as the first BLASTP match on the TAIR database annotated by IPGI are shown. Overlap length (amino acids), percent id and E-value are indicated for each Prunus/Arabidopsis gene pair. Arabidopsis homologues with detectable expression in mature pollen, hydrated pollen and pollen tubes (+/−) and those with altered transcription during pollen germination (PG) or pollen tube growth (PTG) are also indicated according to the results reported by Wang et al. (2008) using Affymetrix ATH1 Genome Arrays. (DOC) [file pone.0053947.s005.doc]

**Table S5 Gene content of the *M’*-locus peach syntenic region.** Position and length of the ORFs as well as the first BLASTP match on the TAIR database annotated by IPGI are shown. Overlap length (amino acids), percent id and E-value are indicated for each *Prunus*/*Arabidopsis* gene pair. *Arabidopsis* homologues with detectable expression in mature pollen, hydrated pollen and pollen tubes (+/-) and those with altered transcription during pollen germination (PG) or pollen tube growth (PTG) are also indicated according to the results reported by Wang et al. (2008) using Affymetrix ATH1 Genome Arrays.

| **Peach Gene ID** | **Transcript**  **Start Stop Length** | | | **TAIR** | **Description** | **Overlap**  **length** | **% id** | **E-value** | **Pollen expr.** | **Altered transc.** |
| --- | --- | --- | --- | --- | --- | --- | --- | --- | --- | --- |
| ppa022538m | 18491554 | 18493033 | 1480 | AT5G15720.1 | GLIP7; carboxylesterase/ lipase | 340 | 44,71 | 1E-76 | - | - |
| ppa006182m | 18495042 | 18498361 | 3320 | AT5G15730.2^b^ | serine/threonine protein kinase, putative | 428 | 66,36 | 3E-155 | - | - |
| ppa002721m | 18498827 | 18500772 | 1946 | AT2G03890.1 | phosphatidylinositol 3- and 4-kinase family protein | 660 | 63,79 | 0 | - | - |
| ppa002370m | 18503945 | 18508593 | 4649 | AT3G30300.1^b^ | unknown protein | 690 | 68,41 | 0 | - | - |
| ppa008856m | 18510031 | 18513228 | 3198 | AT1G69010.1 | BIM2 (BES1-interacting Myc-like protein 2); DNA binding / transcription factor | 273 | 44,32 | 5E-43 | - | - |
| ppa004594m | 18515587 | 18518005 | 2419 | AT5G15740.1 | unknown protein | 508 | 69,09 | 0 | - | - |
| ppa012139m | 18522087 | 18523783 | 1697 | AT5G15750.1^b^ | RNA-binding S4 domain-containing protein | 167 | 80,84 | 1E-80 | - | - |
| ppa000002m | 18524173 | 18545067 | 20895 | AT3G02260.1^b^ | BIG (BIG); binding / ubiquitin-protein ligase/ zinc ion binding | 5008 | 68,11 | 0 | + | / |
| ppa026731m | 18545106 | 18546555 | 1450 | AT3G25270.1 | nucleic acid binding | 149 | 28,86 | 1E-10 | - | - |
| ppa023507m | 18559505 | 18560630 | 1126 | AT4G37180.1 | myb family transcription factor | 52 | 67,31 | 2E-14 | + | downPTG |
| ppa005351m | 18563326 | 18565121 | 1796 | AT5G15780.1 | pollen Ole e 1 allergen and extensin family protein | 168 | 58,93 | 9E-41 | - | - |
| ppa011450m | 18574543 | 18575507 | 965 | N/A | N/A | N/A | N/A | N/A | N/A | N/A |
| ppa001620m | 18581982 | 18588641 | 6660 | AT5G38880.1^b^ | unknown protein | 794 | 74,31 | 0 | - | - |
| ppa011007m | 18590528 | 18594247 | 3720 | AT5G15790.2^b^ | zinc finger (C3HC4-type RING finger) family protein | 231 | 60,61 | 3E-74 | - | - |
| ppa017665m | 18594777 | 18596276 | 1500 | AT5G38900.1 | DSBA oxidoreductase family protein | 207 | 55,07 | 2E-67 | + | / |
| ppa011285m | 18597087 | 18599509 | 2423 | AT5G38900.1 | DSBA oxidoreductase family protein | 214 | 63,08 | 6E-77 | + | / |
| ppa011289m | 18597087 | 18599313 | 2227 | AT5G38900.1 | DSBA oxidoreductase family protein | 214 | 63,08 | 6E-77 | + | / |
| ppa011302m | 18597087 | 18599360 | 2274 | AT5G38900.1^b^ | DSBA oxidoreductase family protein | 214 | 63,08 | 6E-77 | + | / |
| ppa012296m | 18597087 | 18599509 | 2423 | AT5G38900.1 | DSBA oxidoreductase family protein | 213 | 55,4 | 6E-63 | + | / |
| ppa005069m | 18600204 | 18604076 | 3873 | AT3G02300.1^b^ | regulator of chromosome condensation (RCC1) family protein | 463 | 77,97 | 0 | + | / |
| ppa010249m | 18604427 | 18608655 | 4229 | AT1G69120.1 | AP1 (APETALA1); DNA binding / protein binding / protein heterodimerization/ transcription activator/ transcription factor | 152 | 72,37 | 1E-52 | - | - |
| ppa010548m | 18619188 | 18623759 | 4572 | AT3G02310.1 | SEP2 (SEPALLATA 2); DNA binding / protein binding / transcription factor | 251 | 74,9 | 1E-95 | - | - |
| ppa010577m | 18619188 | 18623759 | 4572 | AT3G02310.1^b^ | SEP2 (SEPALLATA 2); DNA binding / protein binding / transcription factor | 250 | 75,2 | 5E-97 | - | - |
| ppa026503m | 18629652 | 18630269 | 618 | AT5G15802.1^b^ | unknown protein | 110 | 56,36 | 9E-30 | - | - |
| ppa016385m | 18630946 | 18633249 | 2304 | AT3G30210.1^b^ | MYB121 (MYB DOMAIN PROTEIN 121); DNA binding / transcription factor | 217 | 51,15 | 2E-52 | - | - |
| ppa013380m | 18636187 | 18636866 | 680 | N/A | N/A | N/A | N/A | N/A | N/A | N/A |
| ppa003386m | 18638891 | 18643879 | 4989 | AT3G02320.1^b^ | RNA binding / tRNA (guanine-N2-)-methyltransferase | 558 | 78,85 | 0 | - | - |
| ppa007756m | 18645262 | 18647647 | 2386 | AT3G02230.1^b^ | RGP1 (REVERSIBLY GLYCOSYLATED POLYPEPTIDE 1); cellulose synthase (UDP-forming) | 337 | 91,1 | 0 | + | / |
| ppa005994m | 18649075 | 18651638 | 2564 | AT3G30180.1^b^ | BR6OX2 (BRASSINOSTEROID-6-OXIDASE 2); monooxygenase/ oxygen binding | 464 | 67,24 | 0 | + | Up PTG |
| ppa007503m | 18660657 | 18662638 | 1982 | AT1G13680.1^b^ | phospholipase C/ phosphoric diester hydrolase | 348 | 62,64 | 1E-129 | - | - |
| ppa014104m | 18664030 | 18664766 | 737 | AT3G29970.1^b^ | germination protein-related | 86 | 69,77 | 5E-34 | + | / |
| ppa024465m | 18666252 | 18668102 | 1851 | AT5G15630.1 | IRX6 | 397 | 80,1 | 0 | - | - |
| ppa005522m | 18668649 | 18671935 | 3287 | AT5G60920.1 | COB (COBRA) | 435 | 74,25 | 0 | - | - |
| ppb023073m | 18676574 | 18678411 | 1838 | AT3G02210.1 | COBL1 (COBRA-LIKE PROTEIN 1 PRECURSOR) | 180 | 42,22 | 1E-33 | - | - |
| ppb020721m | 18704742 | 18706234 | 1493 | AT1G56440.1 | serine/threonine protein phosphatase-related | 56 | 62,5 | 7E-16 | - | - |
| ppa022025m | 18716879 | 18718491 | 1613 | AT2G21660.2 | CCR2 (COLD, CIRCADIAN RHYTHM, AND RNA BINDING 2); RNA binding / double-stranded DNA binding / single-stranded DNA binding | 55 | 65,45 | 5E-15 | - | - |
| ppa007463m | 18748304 | 18751578 | 3275 | AT5G15640.1^b^ | mitochondrial substrate carrier family protein | 319 | 79,31 | 4E-147 | + | Up PTG |
| ppa010178m | 18748304 | 18749962 | 1659 | AT5G15640.1 | mitochondrial substrate carrier family protein | 210 | 80,95 | 2E-98 | + | Up PTG |
| ppa015454m | 18752057 | 18753454 | 1398 | AT4G01240.1 | unknown protein | 329 | 58,66 | 3E-111 | - | - |
| ppa011807m | 18754276 | 18755073 | 798 | AT3G02220.1^b^ | unknown protein | 167 | 69,46 | 7E-63 | - | - |
| ppa013367m | 18755340 | 18756208 | 869 | AT1G24140.1^a^ | Matrixin family protein | 92 | 32 | 7E-6 | + | Up PTG |
| ppa019352m | 18760652 | 18763276 | 2625 | AT3G51550.1 | FER (FERONIA); kinase/ protein kinase | 908 | 45,15 | 0 | - | - |
| ppa001157m | 18766568 | 18769249 | 2682 | AT5G38990.1 | protein kinase family protein | 837 | 46,95 | 1E-175 | - | - |
| ppa001190m | 18774840 | 18777497 | 2658 | AT3G51550.1 | FER (FERONIA); kinase/ protein kinase | 825 | 49,58 | 0 | - | - |
| ppa018922m | 18793680 | 18794048 | 369 | N/A | N/A | N/A | N/A | N/A | N/A | N/A |
| ppa020589m | 18806038 | 18808848 | 2811 | AT3G51550.1 | FER (FERONIA); kinase/ protein kinase | 812 | 51,6 | 0 | - | - |
| ppa013018m | 18809760 | 18810532 | 773 | AT2G33775.1^a^ | RALFL19 ralf-like19 | 231 | 32 | 0.007 | - | - |
| ppa003761m | 18812093 | 18814176 | 2084 | AT3G51550.1 | FER (FERONIA); kinase/ protein kinase | 414 | 44,69 | 1E-84 | - | - |
| ppa001413m | 18815789 | 18818489 | 2701 | AT3G51550.1 | FER (FERONIA); kinase/ protein kinase | 394 | 53,3 | 4E-108 | - | - |
| ppa016279m | 18829367 | 18831840 | 2474 | AT3G51550.1 | FER (FERONIA); kinase/ protein kinase | 815 | 47,48 | 0 | - | - |
| ppa006461m | 18834674 | 18838207 | 3534 | AT5G15610.2^b^ | proteasome family protein | 413 | 68,52 | 3E-177 | - | - |
| ppa017965m | 18839676 | 18840153 | 478 | AT1G69230.2 | SP1L2 (SPIRAL1-LIKE2) | 97 | 48,45 | 1E-14 | - | - |
| ppa007173m | 18841314 | 18844509 | 3196 | AT3G29770.1^b^ | MES11 (METHYL ESTERASE 11); hydrolase | 310 | 77,42 | 1E-125 | - | - |
| ppa007590m | 18849392 | 18852062 | 2671 | AT3G29760.1 | NLI interacting factor (NIF) family protein | 146 | 58,22 | 1E-49 | - | - |
| ppa000735m | 18858946 | 18862792 | 3847 | AT1G74160.1 | unknown protein | 1090 | 35,6 | 1E-116 | - | - |
| ppa007206m | 18863483 | 18864819 | 1337 | AT5G15570.1^b^ | unknown protein  DOMAIN/s: Bromodomain transcription factor  BEST Arabidopsis thaliana protein match is: DNA binding | 391 | 38,87 | 1E-64 | - | - |
| ppa007243m | 18867715 | 18869807 | 2093 | AT3G02150.2^b^ | PTF1 (PLASTID TRANSCRIPTION FACTOR 1); transcription factor | 75 | 88 | 3E-36 | - | - |
| ppa021495m | 18874448 | 18875851 | 1404 | AT3G29635.1 | transferase family protein | 443 | 40,41 | 4E-74 | - | - |
| ppa020932m | 18881667 | 18882581 | 915 | AT1G21280.1^a^ | DOMAIN/s: Retrotransposon gag protein | 294 | 27 | 4E-06 | - | - |
| ppa005255m | 18885375 | 18887152 | 1778 | AT5G39090.1 | transferase family protein | 470 | 37,45 | 2E-78 | - | - |
| ppa026050m | 18893531 | 18896984 | 3454 | AT5G39080.1 | transferase family protein | 436 | 38,99 | 4E-78 | - | - |
| ppa024873m | 18897669 | 18899334 | 1666 | AT3G29635.1 | transferase family protein | 429 | 28,9 | 2E-39 | - | - |
| ppa021452m | 18899747 | 18901162 | 1416 | AT5G39090.1 | transferase family protein | 448 | 41,07 | 9E-85 | - | - |
| ppa022904m | 18911571 | 18913109 | 1539 | AT5G39080.1 | transferase family protein | 431 | 27,61 | 1E-33 | - | - |
| ppa026936m | 18914045 | 18915457 | 1413 | AT5G39090.1 | transferase family protein | 472 | 39,41 | 5E-88 | - | - |
| ppa005502m | 18936429 | 18937883 | 1455 | AT5G39090.1 | transferase family protein | 471 | 39,92 | 3E-85 | - | - |
| ppa020665m | 18940430 | 18941821 | 1392 | AT5G39080.1^b^ | transferase family protein | 465 | 40,43 | 9E-88 | - | - |
| ppa016949m | 18957619 | 18959010 | 1392 | AT5G39080.1 | transferase family protein | 465 | 40,22 | 9E-86 | - | - |
| ppa025189m | 18961426 | 18962349 | 924 | AT5G39090.1 | transferase family protein | 214 | 40,19 | 5E-39 | - | - |
| ppa018052m | 18963290 | 18963619 | 330 | AT5G39080.1 | transferase family protein | 64 | 45,31 | 8E-10 | - | - |
| ppa016023m | 18968998 | 18970547 | 1550 | AT5G39090.1 | transferase family protein | 294 | 32,99 | 1E-31 | - | - |
| ppa005488m | 18972684 | 18974060 | 1377 | AT5G39080.1 | transferase family protein | 468 | 39,53 | 5E-89 | - | - |
| ppa020216m | 18979935 | 18982632 | 2698 | AT5G39080.1 | transferase family protein | 228 | 43,86 | 4E-46 | - | - |
| ppa019904m | 18992254 | 18993632 | 1379 | AT3G29635.1 | transferase family protein | 429 | 38,46 | 1E-72 | - | - |
| ppa016299m | 18995785 | 18997447 | 1663 | AT5G39090.1 | transferase family protein | 477 | 39,41 | 1E-80 | - | - |
| ppa019320m | 18998050 | 18999448 | 1399 | AT5G39080.1 | transferase family protein | 222 | 37,84 | 8E-33 | - | - |
| ppa005348m | 19000743 | 19005122 | 4380 | AT5G60980.2 | nuclear transport factor 2 (NTF2) family protein / RNA recognition motif (RRM)-containing protein | 386 | 48,7 | 8E-83 | - | - |
| ppa007375m | 19007660 | 19009944 | 2285 | AT3G29575.4^b^ | AFP3 (ABI FIVE BINDING PROTEIN 3) | 84 | 82,14 | 5E-38 | + | / |
| ppa000986m | 19011230 | 19014865 | 3636 | AT3G02130.1^b^ | RPK2 (RECEPTOR-LIKE PROTEIN KINASE 2); ATP binding / kinase/ protein serine/threonine kinase | 953 | 67,89 | 0 | - | - |
| ppa009937m | 19018505 | 19020134 | 1630 | AT3G02125.1^b^ | unknown protein | 191 | 33,51 | 1E-12 | + | Up PTG |
| ppa012522m | 19020442 | 19021206 | 765 | AT5G39210.1^b^ | CRR7 (CHLORORESPIRATORY REDUCTION 7) | 162 | 46,91 | 3E-37 | - | - |
| ppa016098m | 19021821 | 19022271 | 451 | AT3G02120.1^b^ | hydroxyproline-rich glycoprotein family protein | 61 | 67,21 | 6E-16 | - | - |
| ppa014248m | 19022519 | 19023590 | 1072 | N/A | N/A | N/A | N/A | N/A | N/A | N/A |
| ppa017321m | 19028160 | 19032318 | 4159 | AT3G02110.1^b^ | scpl25 (serine carboxypeptidase-like 25); serine-type carboxypeptidase | 473 | 74,63 | 0 | - | - |
| ppa005110m | 19034922 | 19036600 | 1679 | AT3G29400.1 | ATEXO70E1 (exocyst subunit EXO70 family protein E1); protein binding | 279 | 50,9 | 3E-71 | - | - |
| ppa004810m | 19038382 | 19044888 | 6507 | AT3G29390.1^b^ | RIK (RS2-Interacting KH protein); RNA binding | 490 | 54,08 | 1E-119 | + | / |
| ppa007817m | 19047717 | 19051663 | 3947 | AT1G13820.1 | hydrolase, alpha/beta fold family protein | 301 | 66,78 | 3E-117 | - | - |
| ppa005967m | 19052928 | 19057394 | 4467 | AT5G15550.1^b^ | transducin family protein / WD-40 repeat family protein | 434 | 72,81 | 0 | - | - |
| ppa000951m | 19058877 | 19065497 | 6621 | AT1G69830.1 | AMY3 (ALPHA-AMYLASE-LIKE 3); alpha-amylase | 403 | 59,8 | 3E-149 | - | - |
| ppa000160m | 19075922 | 19083048 | 7127 | AT5G07980.1 | dentin sialophosphoprotein-related | 412 | 39,81 | 4E-71 | - | - |
| ppa020830m | 19084297 | 19085579 | 1283 | AT3G10330.1 | transcription initiation factor IIB-2 / general transcription factor TFIIB-2 (TFIIB2) | 314 | 50 | 5E-82 | + | / |
| ppa021805m | 19089131 | 19090244 | 1114 | AT3G10330.1 | transcription initiation factor IIB-2 / general transcription factor TFIIB-2 (TFIIB2) | 313 | 45,05 | 2E-68 | + | / |
| ppa024744m | 19091119 | 19092216 | 1098 | AT3G02100.1 | UDP-glucoronosyl/UDP-glucosyl transferase family protein | 264 | 42,05 | 1E-47 | - | - |
| ppa020867m | 19098001 | 19099672 | 1672 | AT3G02100.1 | UDP-glucoronosyl/UDP-glucosyl transferase family protein | 465 | 46,24 | 5E-122 | - | - |
| ppa019116m | 19105243 | 19106720 | 1478 | AT3G02100.1^b^ | UDP-glucoronosyl/UDP-glucosyl transferase family protein | 465 | 47,1 | 3E-123 | - | - |
| ppa020470m | 19107803 | 19109506 | 1704 | AT3G02100.1 | UDP-glucoronosyl/UDP-glucosyl transferase family protein | 464 | 45,47 | 2E-119 | - | - |
| ppa006338m | 19110526 | 19112487 | 1962 | AT3G02100.1 | UDP-glucoronosyl/UDP-glucosyl transferase family protein | 464 | 43,97 | 8E-110 | - | - |
| ppa025965m | 19114187 | 19114441 | 255 | AT5G39240.1^b^ | unknown protein | 90 | 41,11 | 5E-11 | - | - |
| ppa010438m | 19119067 | 19120153 | 1087 | AT5G39250.1^b^ | F-box family protein | 252 | 67,06 | 2E-93 | + | / |
| ppa024239m | 19120527 | 19121460 | 934 | AT5G39300.1 | ATEXPA25 (ARABIDOPSIS THALIANA EXPANSIN A25) | 224 | 65,18 | 2E-89 | - | - |
| ppa000125m | 19123369 | 19135325 | 11957 | AT5G15540.1^b^ | EMB2773 (EMBRYO DEFECTIVE 2773); binding / protein binding / zinc ion binding | 1563 | 67,5 | 0 | - | - |
| ppa009593m | 19136301 | 19138411 | 2111 | AT5G15530.1^b^ | BCCP2 (BIOTIN CARBOXYL CARRIER PROTEIN 2); biotin binding | 289 | 47,06 | 2E-55 | - | - |
| ppa004059m | 19138952 | 19142613 | 3662 | AT3G02090.1^b^ | mitochondrial processing peptidase beta subunit, putative | 480 | 78,75 | 0 | + | / |
| ppa011540m | 19142598 | 19144436 | 1839 | AT5G61170.1 | 40S ribosomal protein S19 (RPS19C) | 139 | 90,65 | 1E-72 | - | - |
| ppa012105m | 19142598 | 19144436 | 1839 | AT5G61170.1 | 40S ribosomal protein S19 (RPS19C) | 120 | 90 | 5E-62 | - | - |
| ppa020426m | 19145946 | 19149339 | 3394 | AT3G01015.1^b^ | unknown protein  DOMAIN/s: Targeting for Xklp2 | 496 | 52,82 | 6E-101 | + | / |
| ppb012981m | 19154167 | 19156382 | 2216 | AT3G26922.1 | unknown protein  DOMAIN/s: Cyclin-like F-box, Leucine-rich repeat 2  BEST Arabidopsis thaliana protein match is: F-box family protein | 286 | 27,27 | 4E-13 | - | - |
| ppa004981m | 19158038 | 19160714 | 2677 | AT5G15490.1 | UDP-glucose 6-dehydrogenase, putative | 482 | 87,55 | 0 | + | / |
| ppa004991m | 19158038 | 19160714 | 2677 | AT5G15490.1 | UDP-glucose 6-dehydrogenase, putative | 482 | 87,55 | 0 | + | / |
| ppa005006m | 19158038 | 19160714 | 2677 | AT5G15490.1 | UDP-glucose 6-dehydrogenase, putative | 482 | 87,55 | 0 | + | / |
| ppa005897m | 19162719 | 19164403 | 1685 | AT1G26580.1 | unknown protein  BEST Arabidopsis thaliana protein match is: myb family transcription factor / ELM2 domain-containing protein | 475 | 32,21 | 1E-43 | + | / |
| ppa016813m | 19167289 | 19168905 | 1617 | AT1G26610.1 | zinc finger (C2H2 type) family protein | 322 | 28,26 | 5E-16 | + | / |
| ppa000827m | 19171126 | 19177833 | 6708 | AT3G29320.1 | glucan phosphorylase, putative | 516 | 74,42 | 0 | - | - |
| ppa010903m | 19180586 | 19181564 | 979 | AT3G29310.1^a^ | Calmodulin-binding protein related | 378 | 29 | 0.023 | - | - |
| ppa023614m | 19182400 | 19185223 | 2824 | AT4G38180.1 | FRS5 (FAR1-related sequence 5); zinc ion binding | 624 | 45,03 | 9E-163 | - | - |
| ppa013993m | 19185732 | 19187433 | 1702 | AT5G61220.1^b^ | complex 1 family protein / LVR family protein | 83 | 55,42 | 1E-22 | - | - |
| ppa014984m | 19187929 | 19189674 | 1746 | AT5G39350.1^b^ | pentatricopeptide (PPR) repeat-containing protein | 583 | 58,15 | 0 | - | - |
| ppa004045m | 19193460 | 19197883 | 4424 | AT5G15470.1^b^ | GAUT14 (Galacturonosyltransferase 14); polygalacturonate 4-alpha-galacturonosyltransferase/ transferase, transferring glycosyl groups / transferase, transferring hexosyl groups | 532 | 87,59 | 0 | + | Down PTG |
| ppa013551m | 19199756 | 19202490 | 2735 | AT3G01050.1^b^ | MUB1 (MEMBRANE-ANCHORED UBIQUITIN-FOLD PROTEIN 1 PRECURSOR) | 117 | 64,1 | 1E-42 | - | - |
| ppa014205m | 19203844 | 19204267 | 424 | N/A | N/A | N/A | N/A | N/A | N/A | N/A |
| ppa007471m | 19205856 | 19207724 | 1869 | N/A | N/A | N/A | N/A | N/A | N/A | N/A |
| ppa000855m | 19208678 | 19213930 | 5253 | AT5G15450.1^b^ | CLPB3 (CASEIN LYTIC PROTEINASE B3); ATP binding / ATPase/ nucleoside-triphosphatase/ nucleotide binding / protein binding | 953 | 85,41 | 0 | + | / |
| ppa017256m | 19214613 | 19216111 | 1499 | AT3G29280.1^b^ | unknown protein | 150 | 75,33 | 9E-59 | - | - |
| ppa010470m | 19216695 | 19219176 | 2482 | AT5G39360.1^b^ | EDL2 (EID1-like 2) | 248 | 83,87 | 7E-126 | - | - |
| ppa007111m | 19220088 | 19221740 | 1653 | AT5G39380.1^b^ | calmodulin-binding protein-related | 275 | 46,55 | 1E-42 | + | / |
| ppa005586m | 19228019 | 19230695 | 2677 | AT3G01060.1^b^ | unknown protein | 455 | 79,78 | 0 | - | - |
| ppa010047m | 19231344 | 19232618 | 1275 | AT3G29270.2^b^ | ubiquitin-protein ligase | 266 | 64,29 | 2E-85 | - | - |
| ppa002126m | 19241079 | 19244334 | 3256 | AT5G15410.1^b^ | DND1 (DEFENSE NO DEATH 1); calcium channel/ calmodulin binding / cation channel/ cyclic nucleotide binding / intracellular cAMP activated cation channel/ intracellular cyclic nucleotide activated cation channel/ inward rectifier potassium channel | 728 | 74,86 | 0 | - | - |
| ppa006437m | 19246165 | 19248673 | 2509 | AT5G39400.1^b^ | PTEN1; phosphatase | 401 | 67,58 | 2E-159 | + | Down PTG |
| ppa018982m | 19249067 | 19251699 | 2633 | AT3G59170.1^a^ | F-box/RNI like superfamily protein | 108 | 62 | 9E-07 | + | / |
| ppa000705m | 19253035 | 19259243 | 6209 | AT5G15400.1^b^ | U-box domain-containing protein | 1042 | 77,26 | 0 | - | - |
| ppa020057m | 19270904 | 19272288 | 1385 | AT1G61500.1 | S-locus protein kinase, putative | 230 | 54,35 | 1E-61 | - | - |
| ppb011385m | 19274222 | 19275245 | 1024 | AT3G47570.1^a^ | Leucine-rich repeat protein kinase family | 174 | 42 | 8E-07 | - | - |
| ppa015373m | 19281903 | 19282828 | 926 | AT2G44970.2 | lipase-related | 168 | 63,69 | 1E-55 | + | / |
| ppa022595m | 19284362 | 19285014 | 653 | AT1G61480.1 | S-locus protein kinase, putative | 113 | 56,64 | 5E-27 | - | - |
| ppa015584m | 19293768 | 19295622 | 1855 | AT5G44940.1^a^ | F-box/RNI like superfamily protein | 492 | 26 | 1E-05 | - | - |
| ppa008118m | 19296139 | 19298487 | 2349 | AT5G15390.1^b^ | tRNA/rRNA methyltransferase (SpoU) family protein | 273 | 72,53 | 2E-114 | - | - |
| ppa027121m | 19299176 | 19300573 | 1398 | AT4G15280.1 | UGT71B5 (UDP-GLUCOSYL TRANSFERASE 71B5); UDP-glycosyltransferase/ quercetin 3-O-glucosyltransferase/ transferase, transferring glycosyl groups | 491 | 31,16 | 6E-49 | - | - |
| ppa015845m | 19304869 | 19306169 | 1301 | AT4G15280.1 | UGT71B5 (UDP-GLUCOSYL TRANSFERASE 71B5); UDP-glycosyltransferase/ quercetin 3-O-glucosyltransferase/ transferase, transferring glycosyl groups | 443 | 32,96 | 8E-45 | - | - |
| ppa005427m | 19307491 | 19308892 | 1402 | AT4G01070.1 | GT72B1; UDP-glucosyltransferase/ UDP-glycosyltransferase/ transferase, transferring glycosyl groups | 477 | 31,24 | 2E-45 | + | Down PG |
| ppa005544m | 19309086 | 19311255 | 2170 | AT5G39410.1^b^ | binding / catalytic | 444 | 71,4 | 1E-170 | - | - |
| ppa001868m | 19311528 | 19313894 | 2367 | AT4G21300.1 | pentatricopeptide (PPR) repeat-containing protein | 664 | 34,49 | 2E-114 | - | - |
| ppa020905m | 19318081 | 19318432 | 352 | AT3G07490.1 | AGD11 (ARF-GAP domain 11); calcium ion binding | 54 | 62,96 | 2E-14 | + | Up PG/PTG |
| ppa013269m | 19328280 | 19329440 | 1161 | N/A | N/A | N/A | N/A | N/A | N/A | N/A |
| ppa025951m | 19355024 | 19356771 | 1748 | N/A | N/A | N/A | N/A | N/A | N/A | N/A |
| ppa022986m | 19372463 | 19374770 | 2308 | N/A | N/A | N/A | N/A | N/A | N/A | N/A |
| ppa018209m | 19378653 | 19379885 | 1233 | AT1G13940.1 | unknown protein | 275 | 30,18 | 2E-10 | - | - |
| ppa026630m | 19387745 | 19390960 | 3216 | AT1G26620.1 | unknown protein | 934 | 26,45 | 4E-43 | - | - |
| ppa010430m | 19396610 | 19397388 | 779 | AT3G29240.2^b^ | unknown protein | 237 | 71,31 | 5E-89 | - | - |
| ppa017633m | 19398358 | 19400169 | 1812 | AT3G29230.1^b^ | pentatricopeptide (PPR) repeat-containing protein | 570 | 64,56 | 0 | - | - |
| ppa008650m | 19401227 | 19404376 | 3150 | AT3G29200.1^b^ | CM1 (CHORISMATE MUTASE 1); L-ascorbate peroxidase/ chorismate mutase | 340 | 62,06 | 3E-107 | - | - |
| ppa012248m | 19405752 | 19407075 | 1324 | AT5G15350.1^b^ | plastocyanin-like domain-containing protein | 151 | 57,62 | 3E-41 | - | - |
| ppa022822m | 19407697 | 19409782 | 2086 | AT5G15870.1 | glycosyl hydrolase family 81 protein | 670 | 60,9 | 0 | + | Up PTG |
| ppa020122m | 19410871 | 19411919 | 1049 | AT2G27035.1 | plastocyanin-like domain-containing protein | 112 | 45,54 | 3E-28 | - | - |
| ppa017224m | 19412641 | 19413914 | 1274 | AT1G69390.1 | ATMINE1 (Arabidopsis homologue of bacterial MinE 1); protein binding | 215 | 54,88 | 9E-55 | - | - |
| ppa006467m | 19414851 | 19417373 | 2523 | AT3G29185.1^b^ | unknown protein | 382 | 68,85 | 9E-152 | - | - |
| ppa003809m | 19419766 | 19423127 | 3362 | AT1G13960.1 | WRKY4; DNA binding / transcription factor | 478 | 52,72 | 4E-107 | + | Up PTG |
| ppa002153m | 19425256 | 19428803 | 3548 | AT5G39420.1^b^ | cdc2cAt (Arabidopsis thaliana cdc2c); ATP binding / kinase/ protein kinase/ protein serine/threonine kinase | 602 | 53,99 | 0 | - | - |
| ppa003330m | 19430187 | 19433082 | 2896 | AT3G29180.1^b^ | unknown protein | 489 | 62,99 | 2E-176 | + | / |
| ppa005577m | 19444944 | 19447021 | 2078 | AT3G29180.1 | unknown protein | 358 | 49,16 | 1E-84 | + | / |
| ppa019328m | 19452185 | 19454055 | 1871 | AT1G61420.1 | S-locus lectin protein kinase family protein | 180 | 51,11 | 2E-37 | - | - |
| ppa024828m | 19455075 | 19455374 | 300 | AT1G48940.1 | plastocyanin-like domain-containing protein | 57 | 68,42 | 7E-19 | - | - |
| ppa013579m | 19460383 | 19462427 | 2045 | AT3G29170.1^b^ | unknown protein | 121 | 68,6 | 5E-30 | + | Up PTG |
| ppa004347m | 19463421 | 19468043 | 4623 | AT3G01090.2^b^ | AKIN10 (Arabidopsis SNF1 kinase homolog 10); protein binding / protein kinase | 498 | 86,35 | 0 | + | / |
| ppa025010m | 19468943 | 19470217 | 1275 | AT5G19790.1 | RAP2.11 (related to AP2 11); DNA binding / transcription factor | 77 | 76,62 | 1E-19 | - | - |
| ppa002110m | 19474159 | 19480251 | 6093 | AT3G01100.1^b^ | HYP1 (HYPOTHETICAL PROTEIN 1) | 701 | 62,91 | 0 | + | / |
| ppa019884m | 19483949 | 19489940 | 5992 | AT3G01100.1 | HYP1 (HYPOTHETICAL PROTEIN 1) | 747 | 60,91 | 0 | + | / |
| ppa011182m | 19491797 | 19494720 | 2924 | AT5G39510.1^b^ | SGR4 (SHOOT GRAVITROPSIM 4); receptor | 219 | 73,52 | 3E-87 | + | / |
| ppa020933m | 19494965 | 19496866 | 1902 | AT5G15340.1^b^ | pentatricopeptide (PPR) repeat-containing protein | 628 | 58,92 | 0 | - | - |
| ppa010375m | 19497382 | 19498954 | 1573 | AT5G39530.1^b^ | unknown protein | 257 | 40,08 | 5E-47 | - | - |
| ppa008873m | 19499720 | 19502738 | 3019 | AT3G29090.1^b^ | pectinesterase family protein | 312 | 80,77 | 5E-151 | - | - |
| ppb013941m | 19503679 | 19505433 | 1755 | AT3G29075.1 | glycine-rich protein | 98 | 51,02 | 1E-17 | + | / |
| ppa005604m | 19505477 | 19508028 | 2552 | AT3G29075.1^b^ | glycine-rich protein | 141 | 51,06 | 4E-23 | + | / |
| ppa004696m | 19508444 | 19510885 | 2442 | AT1G74630.1 | pentatricopeptide (PPR) repeat-containing protein | 507 | 37,28 | 4E-98 | - | - |
| ppb009532m | 19510291 | 19512202 | 1912 | AT1G14010.1 | emp24/gp25L/p24 family protein | 204 | 63,73 | 3E-61 | - | - |
| ppa001662m | 19513691 | 19518100 | 4410 | AT3G29060.1^b^ | unknown protein  DOMAIN/s: EXS, C-terminal, SPX, N-terminal | 824 | 58,01 | 0 | + | Down PTG |
| ppa008419m | 19518793 | 19520882 | 2090 | AT5G15330.1^b^ | SPX4 (SPX DOMAIN GENE 4) | 305 | 65,57 | 3E-96 | - | - |
| ppa004232m | 19522035 | 19525797 | 3763 | AT3G01120.1^b^ | MTO1 (METHIONINE OVERACCUMULATION 1); cystathionine gamma-synthase | 394 | 87,82 | 0 | + | Up PTG |
| ppa006769m | 19536562 | 19538472 | 1911 | AT5G15310.1^b^ | ATMYB16 (MYB DOMAIN PROTEIN 16); DNA binding / transcription factor | 396 | 53,54 | 5E-91 | - | - |
| ppa007883m | 19548346 | 19549836 | 1491 | AT5G61430.1 | ANAC100 (ARABIDOPSIS NAC DOMAIN CONTAINING PROTEIN 100); transcription factor | 363 | 57,3 | 2E-103 | - | - |
| ppa003946m | 19554779 | 19556419 | 1641 | AT5G15300.1^b^ | pentatricopeptide (PPR) repeat-containing protein | 543 | 60,22 | 0 | - | - |
| ppa013251m | 19560340 | 19561689 | 1350 | N/A | N/A | N/A | N/A | N/A | N/A | N/A |
| ppa014933m | 19562764 | 19563770 | 1007 | AT5G15300.1 | pentatricopeptide (PPR) repeat-containing protein | 257 | 42,02 | 2E-47 | - | - |
| ppa018277m | 19564757 | 19566145 | 1389 | AT5G49610.1^a^ | F-box family | 744 | 23 | 1E-04 | + | / |
| ppa021574m | 19567105 | 19570089 | 2985 | AT5G15280.1^b^ | pentatricopeptide (PPR) repeat-containing protein | 978 | 46,63 | 0 | - | - |
| ppa014463m | 19575091 | 19575831 | 741 | N/A | N/A | N/A | N/A | N/A | N/A | N/A |
| ppa003758m | 19576121 | 19579601 | 3481 | AT5G15270.1^b^ | KH domain-containing protein | 480 | 67,08 | 2E-160 | - | - |
| ppa004961m | 19583491 | 19587280 | 3790 | AT3G01150.1^b^ | PTB1 (POLYPYRIMIDINE TRACT-BINDING PROTEIN 1); RNA binding / nucleic acid binding / nucleotide binding | 349 | 85,96 | 0 | - | - |
| ppa010654m | 19591405 | 19592794 | 1390 | AT2G03090.1 | ATEXPA15 (ARABIDOPSIS THALIANA EXPANSIN A15) | 227 | 80,62 | 9E-77 | - | - |
| ppa015390m | 19595392 | 19597506 | 2115 | AT5G39680.1^b^ | EMB2744 (EMBRYO DEFECTIVE 2744) | 688 | 54,22 | 0 | - | - |
| ppa011701m | 19598419 | 19599223 | 805 | AT5G39670.1^b^ | calcium-binding EF hand family protein | 203 | 48,77 | 4E-38 | - | - |
| ppa014215m | 19600973 | 19601914 | 942 | N/A | N/A | N/A | N/A | N/A | N/A | N/A |
| ppa004451m | 19604860 | 19608044 | 3185 | AT5G39660.2^b^ | CDF2 (CYCLING DOF FACTOR 2); DNA binding / protein binding / transcription factor | 527 | 43,64 | 2E-81 | + | / |
| ppa010896m | 19611500 | 19612889 | 1390 | AT3G01170.1^b^ | structural constituent of ribosome | 224 | 59,82 | 4E-64 | - | - |
| ppa002164m | 19614676 | 19616973 | 2298 | AT5G50390.1^b^ | pentatricopeptide (PPR) repeat-containing protein | 714 | 61,9 | 0 | - | - |
| ppb020889m | 19617307 | 19617753 | 447 | AT1G26800.1 | zinc finger (C3HC4-type RING finger) family protein | 47 | 55,32 | 0,0000001 | - | - |
| ppa008635m | 19623160 | 19626937 | 3778 | AT3G28970.1^b^ | AAR3 (antiauxin-resistant 3) | 304 | 54,28 | 9E-80 | + | Down PTG |
| ppa001734m | 19627228 | 19632018 | 4791 | AT3G01180.1_b_ | AtSS2 (starch synthase 2); transferase, transferring glycosyl groups | 742 | 68,19 | 0 | - | - |
| ppa020648m | 19633174 | 19633872 | 699 | AT1G09157.1^b^ | unknown protein | 187 | 71,12 | 2E-70 | - | - |
| ppa002364m | 19634693 | 19637215 | 2523 | AT5G15250.1^b^ | FTSH6 (FTSH PROTEASE 6); ATP-dependent peptidase/ ATPase/ metallopeptidase/ peptidase/ zinc ion binding | 651 | 78,8 | 0 | + | / |
| ppa004522m | 19637658 | 19639175 | 1518 | AT2G36730.1^b^ | pentatricopeptide (PPR) repeat-containing protein | 489 | 62,58 | 0 | - | - |
| ppa006034m | 19639635 | 19641354 | 1720 | AT3G28960.1^b^ | amino acid transporter family protein | 405 | 62,96 | 2E-144 | - | - |
| ppa016771m | 19647980 | 19650114 | 2135 | AT5G37820.1 | NIP4;2 (NOD26-LIKE INTRINSIC PROTEIN 4;2); water channel | 241 | 48,96 | 1E-58 | - | - |
| ppa000362m | 19650301 | 19659117 | 8817 | AT5G48600.1^b^ | ATSMC3 (ARABIDOPSIS THALIANA STRUCTURAL MAINTENANCE OF CHROMOSOME 3); ATP binding / transporter | 1238 | 74,88 | 0 | - | - |
| ppa016732m | 19661696 | 19662608 | 913 | AT2G02960.5 | zinc finger (C3HC4-type RING finger) family protein | 80 | 55 | 3E-22 | + | / |
| ppa013692m | 19664452 | 19665301 | 850 | AT5G15230.1^b^ | GASA4 (GAST1 PROTEIN HOMOLOG 4) | 92 | 68,48 | 3E-21 | - | - |
| ppa014185m | 19665910 | 19666850 | 941 | N/A | N/A | N/A | N/A | N/A | N/A | N/A |
| ppa008908m | 19674589 | 19675917 | 1329 | AT3G28920.1^b^ | AtHB34 (ARABIDOPSIS THALIANA HOMEOBOX PROTEIN 34); DNA binding / transcription factor | 256 | 52,73 | 6E-48 | - | - |
| ppa013998m | 19683618 | 19684281 | 664 | AT3G28917.1^b^ | MIF2 (MINI ZINC FINGER 2); DNA binding | 99 | 69,7 | 7E-22 | - | - |
| ppa008366m | 19691186 | 19693083 | 1898 | AT3G28910.1^b^ | MYB30 (MYB DOMAIN PROTEIN 30); DNA binding / transcription factor | 361 | 51,8 | 4E-87 | - | - |
| ppa014261m | 19703691 | 19703931 | 241 | N/A | N/A | N/A | N/A | N/A | N/A | N/A |
| ppb011184m | 19705415 | 19707177 | 1763 | AT1G26880.1^b^ | 60S ribosomal protein L34 (RPL34A) | 95 | 95,79 | 1E-48 | + | / |
| ppa005314m | 19707885 | 19711622 | 3738 | AT5G39830.1^b^ | DEG8; peptidase/ serine-type peptidase | 447 | 74,72 | 0 | - | - |
| ppa016710m | 19715699 | 19717197 | 1499 | AT1G26870.1 | FEZ (FEZ); transcription factor | 216 | 71,76 | 5E-82 | - | - |
| ppa001533m | 19717958 | 19720378 | 2421 | AT5G39840.1^b^ | ATP-dependent RNA helicase, mitochondrial, putative | 808 | 64,73 | 0 | - | - |
| ppa017567m | 19721911 | 19727462 | 5552 | AT3G28880.1^b^ | protein binding | 410 | 46,1 | 9E-66 | - | - |
| ppa010320m | 19731857 | 19735527 | 3671 | AT1G18800.1 | NRP2 (NAP1-RELATED PROTEIN 2); DNA binding / chromatin binding / histone binding | 226 | 61,5 | 1E-74 | - | - |
| ppa000039m | 19735991 | 19744143 | 8153 | AT3G55160.1^b^ | unknown protein  DOMAIN/s: HEAT | 2217 | 58,86 | 0 | - | - |
| ppa001982m | 19750021 | 19753347 | 3327 | AT1G69670.1 | CUL3B (CULLIN 3B); protein binding / ubiquitin-protein ligase | 733 | 81,99 | 0 | + | / |
| ppa011733m | 19756541 | 19758934 | 2394 | AT5G39850.1^b^ | 40S ribosomal protein S9 (RPS9C) | 179 | 92,74 | 5E-96 | - | - |
| ppa027069m | 19759943 | 19760335 | 393 | AT5G15190.2^b^ | unknown protein | 98 | 41,84 | 0,00000001 | - | - |
| ppa005626m | 19761253 | 19763026 | 1774 | AT1G50010.1 | TUA2; structural constituent of cytoskeleton | 435 | 98,39 | 0 | + | / |
| ppa000359m | 19767597 | 19774531 | 6935 | AT3G28860.1^b^ | ABCB19; ATPase, coupled to transmembrane movement of substances / auxin efflux transmembrane transporter | 1247 | 90,3 | 0 | - | - |

^a^ BLASTP TAIR matches with an E-value >1e-6 not included by IPGI (E-value <0.05)

^b^ Gene pairs that are ‘best-reciprocal BLASTP hits’ between *Prunus* and *Arabidopsis*
