# Supplementary material for: An S-Locus Independent Pollen Factor Confers Self-Compatibility in ‘Katy’ Apricot
Source: PLoS One. 2013 Jan 14;8(1):e53947. doi: 10.1371/journal.pone.0053947 (PMC3544744; doi:10.1371/journal.pone.0053947)
Supplement: Table S6 — Primers used in this study to amplify by PCR different fragments corresponding to S-RNase , SFB and actin genes. (DOC) [file pone.0053947.s006.doc]

**Table S6 Primers used in this study to amplify by PCR different fragments corresponding to *S-RNase*, *SFB* and *actin* genes.**

| Primer | Sequence | Reference |
| --- | --- | --- |
| SRc-F | 5’-CTC GCT TTC CTT GTT CTT GC-3’ | Romero *et al*. (2004) |
| SRc-R | 5’-GGC CAT TGT TGC ACA AAT TG-3’ | Romero *et al*. (2004) |
| PruC2 | 5’-CTT TGG CCA AGT AAT TAT TCA AAC C-3’ | Tao *et al*. (1999) |
| PruC2R | 5’-GGT TTG AAT AAT TAC TTG GCC ATA G-3’ | Tao *et al*. (1999) |
| PruC4R | 5’-GGA TGT GGT ACG ATT GAA GCG-3’ | Tao *et al*. (1999) |
| FBf-Hap1 | 5’-TGG AAG CAC CAA TTT ATT TCC T-3` | This work |
| FBr-Hap1 | 5’-TGA TTG AAG GAT CGA TCA TCT TGG-3’ | This work |
| FBf-Hap2 | 5´-GCC CAA TTA CTT GGT CAC TG-3´ | Vilanova *et al*. (2006) |
| FBr-Hap2 | 5´-CAC CCA CTT GAC TTG TCA GC-3´ | Vilanova *et al*. (2006) |
| RT-SFB1-for | 5´-GGC AGC TCG AGT TTT GTT AGC ATA C-3´ | This work |
| RT-SFB1-rev1 | 5´-GGA ACC CGA ATT GGA GAG AAA CGA G-3´ | This work |
| RT-SFB2-for | 5´-TTG GCA GCT CAA GTT TTG TTA GTG C-3´ | This work |
| RT-SFB2-rev2 | 5´-GCA GAA CCC ATA AGT CAG CTT TTC G-3´ | This work |
| Act3 | 5´-CTT CTT ACT GAG GCA CCC CTG AAT-3´ | Gabino Ríos personal comm. |
| Act4 | 5´-AGC ATA GAG GGA GAG AAC TGC TTG-3´ | Gabino Ríos personal comm. |
