# Supplementary material for: An S-Locus Independent Pollen Factor Confers Self-Compatibility in ‘Katy’ Apricot
Source: PLoS One. 2013 Jan 14;8(1):e53947. doi: 10.1371/journal.pone.0053947 (PMC3544744; doi:10.1371/journal.pone.0053947)
Supplement: Table S7 — SSR markers tested for the PPM screening on the whole ‘Katy’ genome. (DOC) [file pone.0053947.s007.doc]

**Table S7 SSR markers tested for the PPM screening on the whole ‘Katy’ genome.**

| Acronyme | Species | Number of SSR | Reference |
| --- | --- | --- | --- |
| BPPCT | *P. persica* | 7 (4)^a^ | Dirlewanger *et al*. (2002) |
| Gol | *P. armeniaca* | 2 (1) | Vera-Ruiz *et al*. (2010) |
| CPDCT | *P. dulcis* | 2 (2) | Mnejja *et al*. (2005) |
| CPPCT | *P. persica* | 5 (4) | Aranzana *et al*. (2002) |
| CPSCT | P. salicina | 14 (6) | Mnejja *et al*. (2004) |
| EPPCU/EPDCU | *P. persica*/*P. dulcis* | 10 (5) | Howad *et al*. (2005), http://www.rosaceae.org/ |
| M/MA | *P. persica* | 7 (3) | Yamamoto *et al*. (2002) |
| pchcms/pchgms | *P. persica* | 2 (1) | Sosinski *et al*. (2000) |
| SsrPaCITA | *P. armeniaca* | 8 (3) | Lopes *et al*. (2002) |
| UDAp | *P. armeniaca* | 20 (10) | Messina *et al*. (2004), http://www.rosaceae.org/ |
| UDP | *P. persica* | 6 (3) | Cipriani *et al*. (1999) and Testolin *et al*. (2000) |
| UDA | *P. dulcis* | 4 (0) | Testolin *et al.* (2004) |
| AMPA | *P. armeniaca* | 1 (1) | Hagen et al. (2004) |
| UCD-CH | *P. avium* | 1 (1) | Struss et al. (2003) |
| PGS | *P. persica* | 29 (11) | Zuriaga et al. (2012) |
| TOTAL |  | 118 (55) |  |

^a^ Number of polymorphic SSRs in ‘Katy’ is indicated between brackets.

**References**

Aranzana MJ, Carbo J, Arus P. (2002) Microsatellite variability in peach [*Prunus persica* (L.) Batsch]: cultivar identification, marker mutation, pedigree inferences and population structure. Theor Appl Genet 106: 1341–1352.

Cipriani G, Lot G, Huang WG, Marrazzo MT, Peterlunger E, et al. (1999) AC/GT and AG/CT microsatellite repeats in peach [*Prunus persica* (L) Batsch]: isolation, characterisation and cross species amplification in Prunus. Theor Appl Genet 99: 65–72.

Dirlewanger E, Cosson P, Tavaud M, Aranzana J, Poizat C, et al. (2002) Development of microsatellite markers in peach [*Prunus persica* (L.) Batsch] and their use in genetic diversity analysis in peach and sweet cherry (*Prunus avium* L.). Theor Appl Genet 105: 127-138.

Hagen LS, Chaib J, Fady B, Decroocq V, Bouchet JP, et al. (2004) Genomic and cDNA microsatellites from apricot (*Prunus armeniaca* L.). Mol Ecol Notes 4: 742–745.

Howad W, Yamamoto T, Dirlewanger E, Testolin R, Cosson P, et al. (2005) Mapping with a few plants: using selective mapping for microsatellite saturation of the Prunus reference map. Genetics 171: 1305–1309.

Lopes MS, Sefc KM, Laimer M, da Camara Machado A (2002) Identification of microsatellite loci in apricot. Mol Ecol Notes 2: 24–26.

Messina R, Lain O, Marrazzo MT, Cipriani G, Testolin R (2004) New set of microsatellite loci isolated in apricot. Mol Ecol Notes 4: 432–434.

Mnejja M, García-Mas J, Howad W, Badenes L, Arus P (2004) Simple-sequence repeat (SSR) markers of Japanese plum (*Prunus salicina* Lindl.) are highly polymorphic and transferable to peach and almond. Mol Ecol Notes 4: 163-166.

Mnejja M, García-Mas J, Howad W, Arus P (2005) Development and transportability across *Prunus* species of 42 polymorphic almond microsatellites. Mol Ecol Notes 5: 531-535.

Sosinski B, Gannavarapu M, Hager LD, Beck LE, King GJ, et al. (2000) Characterization of microsatellite markers in peach (*Prunus persica* L. Batsch). Theor Appl Genet 101: 421–424.

Struss D, Ahmad R, Southwick SM, Boritzki M (2003) Analysis of sweet analysis of sweet cherry (*Prunus avium* L.) cultivars using SSR and AFLP markers. J Am Sic Hort Sci 128: 904-909.

Testolin R, Marrazzo MT, Cipriani G, Quarta R, Verde I, et al. (2000) Microsatellite DNA in peach (*Prunus persica* L. Batsch) and its use in fingerprinting and testing the genetic origin of cultivars. Genome 43: 512–520.

Testolin R, Messina R, Lain O, Marrazzo MT, Huang WG, et al. (2004) Microsatellites isolated in almond from an AC-repeat enriched library. Mol Ecol Notes 4: 459–461

Vera-Ruiz EM, Soriano JM, Romero C, Zhebentyayeva T, Terol J, et al. (2010) Narrowing down the apricot *plum pox virus* resistance locus and comparative analysis with the peach genome syntenic region. Mol Plant Pathol 12: 535-47.

Yamamoto T, Mochida K, Imai T, Shi YZ, Ogiwara I, et al. (2002) Microsatellite markers in peach [*Prunus persica* (L.) Batsch] derived from an enriched genomic and cDNA libraries. Mol Ecol Notes 23: 298-301.

Zuriaga E , Molina L, Badenes ML, Romero C (2012) Physical mapping of a pollen modifier locus controlling self-incompatibility in apricot and synteny analysis within the Rosaceae. Plant Mol Biol 79: 229–242.
